# Electrochemical Activation of Natural Magnesite for Phosphorus Recovery from Wastewater via In Situ Struvite Crystallization

**DOI:** 10.3390/molecules31173047

**Published:** 2026-08-30

**Authors:** Zepeng Wang, Peidong Su, Chunhui Zhang, Weilong Zhou, Xiaoping Zhong, Guifeng Zhao, Zaining Li, Bingxu Quan

**Affiliations:** School of Chemical & Environmental Engineering, China University of Mining & Technology (Beijing), Beijing 100083, China; 15130208699@163.com (Z.W.); zhou138104@163.com (W.Z.); zxp15779091097@163.com (X.Z.); zgfwor@163.com (G.Z.); zainingli@163.com (Z.L.); 15030531019@163.com (B.Q.)

**Keywords:** phosphorus recovery, struvite crystallization, electrochemical struvite precipitation, magnesium carbonate, wastewater treatment, response surface methodology

## Abstract

Phosphorus recovery from wastewater can reduce dependence on phosphate rock and help control eutrophication. Struvite crystallization is a promising recovery route, but conventional processes often require soluble magnesium salts and alkaline reagents. This study used electrochemical activation of natural magnesite to supply Mg^2+^ in situ for magnesium ammonium phosphate (MAP) formation. Magnesite dissolution provided Mg^2+^, while cathodic water reduction generated a local alkaline zone that promoted phosphate precipitation. The effects of current density, N:P molar ratio, initial phosphorus concentration and initial pH were examined. XRD, SEM–EDS, and FTIR supported the formation of struvite-containing precipitates. Under the optimized conditions, namely an initial pH of 7.75, a nominal anodic current density of 28.21 A/m^2^, an initial phosphorus concentration of 6.41 mM, and an N:P molar ratio of 1.42:1, PO_4_^3−^–P recovery, NH_4_^+^––N apparent removal and apparent MAP purity reached 57.56%, 52.26% and 62.56%, respectively. The apparent MAP purity represents an NH_4_^+^––N-based operational estimate rather than an absolute quantitative phase fraction. The decrease in aqueous NH_4_^+^––N should be regarded as apparent removal because NH_3_ volatilization or stripping may also occur near the alkaline cathode. This work provides a reagent-saving approach for phosphorus recovery by coupling Mg^2+^ release, cathodic alkalization and struvite crystallization.

## 1. Introduction

Phosphorus is a key element for agricultural production and biological metabolism, but phosphate rock remains a finite and unevenly distributed resource. Its long-term supply is increasingly affected by rising demand and inefficient use along the food-production chain [1,2]. At the same time, phosphorus-rich effluents from agricultural, industrial and municipal sources continue to increase the nutrient load entering aquatic environments. Excess phosphorus can induce eutrophication, algal blooms, lower water transparency and dissolved oxygen depletion, ultimately affecting aquatic ecosystem structure and function [3,4,5,6]. Recovering phosphorus from wastewater is therefore relevant to both pollution control and phosphorus circularity. It has been estimated that effective phosphorus recovery from wastewater could substitute approximately 20–22% of global phosphorus consumption.

Phosphorus removal from wastewater is commonly achieved through physicochemical and biological approaches, including adsorption or sorptive media, chemical precipitation, ion exchange, and biological treatment [7,8,9,10,11]. Electrochemical processes have also emerged as promising routes for phosphorus removal and recovery because phosphate enrichment, pH regulation, and mineral precipitation can be integrated within the same system [11,12,13,14]. Among these approaches, sacrificial Mg anodes can supply Mg^2+^ in situ, although anode composition, applied current, and surface passivation may affect Mg dissolution and struvite formation [15].

Adsorption is suitable for low-concentration phosphorus-containing wastewater, whereas biological approaches rely on polyphosphate-accumulating organisms under alternating aerobic and anaerobic conditions but are sensitive to operation and require longer treatment times [10,11]. Precipitation-based processes are operationally simpler and compatible with different magnesium sources, although their performance is limited by pH control, reagent consumption and competing by-products [16,17].

Among precipitation routes, struvite crystallization is widely studied because it can recover phosphate and immobilize ammonium as magnesium ammonium phosphate, a slow-release fertilizer with agricultural value [13,18]. Recovered struvite has also been explored for use in construction materials and adsorbents [19]. These characteristics make struvite crystallization suitable for high-nitrogen and high-phosphorus wastewaters, including swine wastewater, anaerobic digestate and landfill leachate.

Struvite crystallization is mainly controlled by solution pH, the molar balance of reactive ions and the magnesium source. These variables determine supersaturation, nucleation and product purity [20,21]. Although pH 9.0–10.0 is generally favorable for phosphorus recovery, the struvite formation process produces acid that reacts with the alkalinity in the bulk solution. In conventional practice, alkaline reagents such as NaOH or Ca(OH)_2_ are often required to maintain suitable conditions. However, in an electrochemical system, OH^−^ ions are continuously generated at the cathode, which can effectively compensate for the consumed alkalinity and reduce the need for external chemical inputs.

The theoretical Mg:N:P molar ratio for struvite formation is 1:1:1, but magnesium salts are commonly overdosed in engineering applications to improve phosphorus recovery, with Mg/P ratios often adjusted to 1.2–1.4. Coexisting Ca^2+^ can also compete with phosphate and form calcium phosphate phases such as hydroxyapatite, lowering struvite purity and recovery efficiency. In addition to alkaline reagents, the magnesium source is a major cost contributor, accounting for approximately 75% of total production costs.

MgCl_2_ and Mg(OH)_2_ are commonly used magnesium sources, but their cost remains a practical limitation. Seawater and industrial brine can reduce magnesium input costs, yet their use depends on local availability. Magnesite (MgCO_3_), an abundant and low-cost magnesium-bearing mineral, is a potential substitute for soluble magnesium salts [22]. Its direct use, however, is restricted by low solubility and weak reactivity in neutral aqueous systems. Electrochemical activation can address this limitation by separating the functions of Mg^2+^ release and alkalinity generation: anodic acidification accelerates magnesite dissolution, whereas cathodic alkalization favors struvite crystallization near the cathode interface [23,24,25]. Regulating Mg^2+^ release and local alkalinity in situ may therefore reduce chemical addition in struvite-based phosphorus recovery.

This work focused on coupling electrochemical magnesite activation with struvite crystallization for phosphorus recovery and simultaneous NH_4_^+^–N apparent removal from wastewater. The specific objectives were to: (1) evaluate the feasibility of using natural magnesite as an electrochemically activated Mg source; (2) examine the effects of current density, initial N:P molar ratio, initial phosphorus concentration and initial pH on PO_4_^3−^–P recovery, NH_4_^+^––N apparent removal and apparent MAP purity, followed by process optimization using response surface methodology (RSM); and (3) provide a qualitative mechanistic interpretation of the coupled roles of anodic magnesite dissolution, cathodic alkalization, ion transport, and the development of cathode-adjacent conditions favorable for heterogeneous struvite crystallization. The study was intended to define the reaction pathway and operating window of a mineral-based, low-chemical phosphorus recovery system.

## 2. Results and Discussion

### 2.1. Characterization of Raw Magnesite and Feasibility of the Electrochemical System

**Figure 1:**
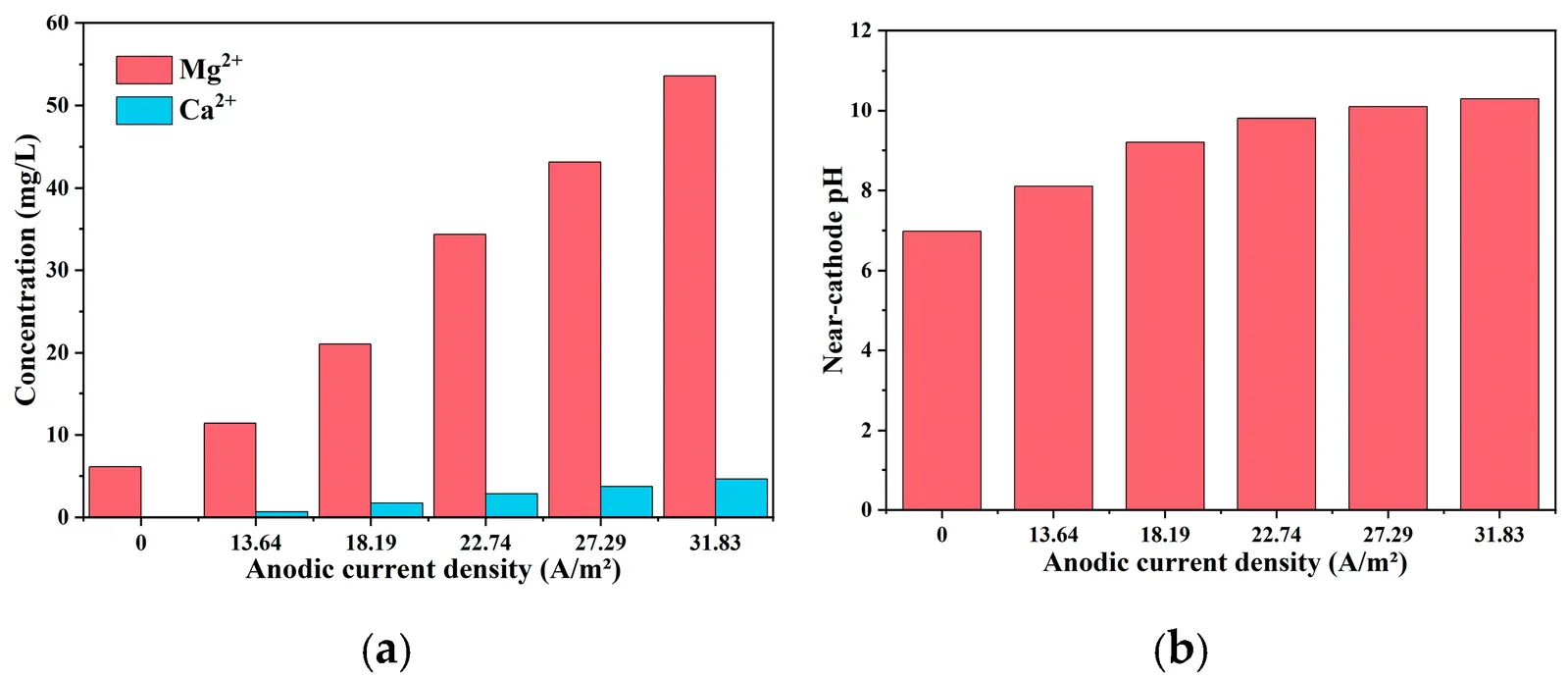
Effects of applied current density on (**a**) Ca^2+^ and Mg^2+^ concentrations in solution and (**b**) pH near the cathode.

The feasibility of coupling electrochemical treatment with magnesite was first evaluated by examining Mg^2+^ release and cathodic alkalinity generation under different current densities. As shown in Figure 1a, the Mg^2+^ concentration was only 6.1 mg/L without an applied current but increased to 53.58 mg/L when the current density reached 31.83 A/m^2^. This increase indicates that electrochemical polarization promoted magnesite dissolution and enhanced Mg^2+^ release from the mineral. In contrast, the concentration of Ca^2+^ released from the raw material remained low (<4.7 mg/L), and the Ca/Mg molar ratio was well below 1:10, suggesting that Ca^2+^ competition during subsequent struvite crystallization was limited [20].

The system also generated an alkaline microenvironment near the cathode. As shown in Figure 1b, the cathode-adjacent pH increased with current density and reached 10.3 at 31.83 A/m^2^, within the alkaline range generally favorable for struvite crystallization (8.0–10.5). In the nitrogen- and phosphorus-containing system, the buffering effects of NH_4_^+^ and phosphate species slightly lowered the pH, but the final pH still remained at 9.0–10.2. The presence of nitrogen and phosphorus also promoted Mg^2+^ dissolution, indicating that the reaction environment was suitable for coupling Mg^2+^ release with phosphate precipitation. These cathode-adjacent pH values represent measurements made in the solution near the cathode and do not resolve the microscale interfacial pH within the cathodic diffusion layer.

**Figure 2:**
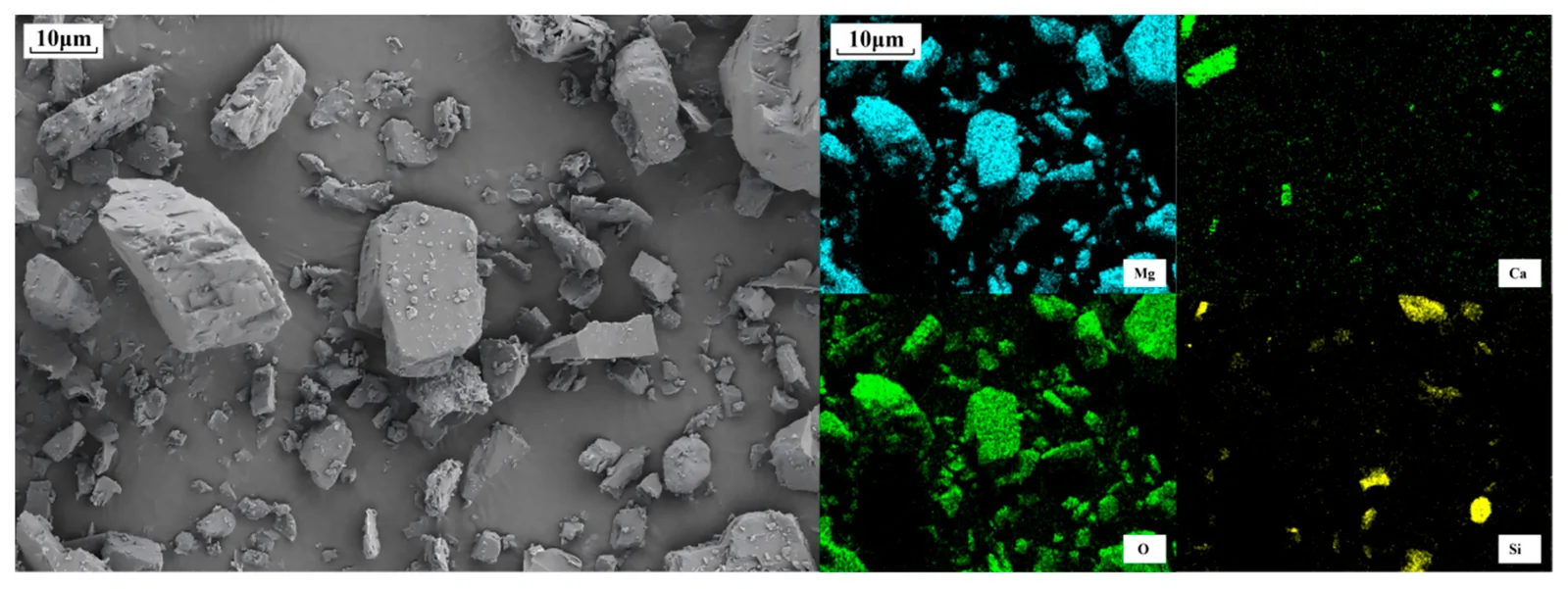
SEM image of raw magnesite.

**Figure 3:**
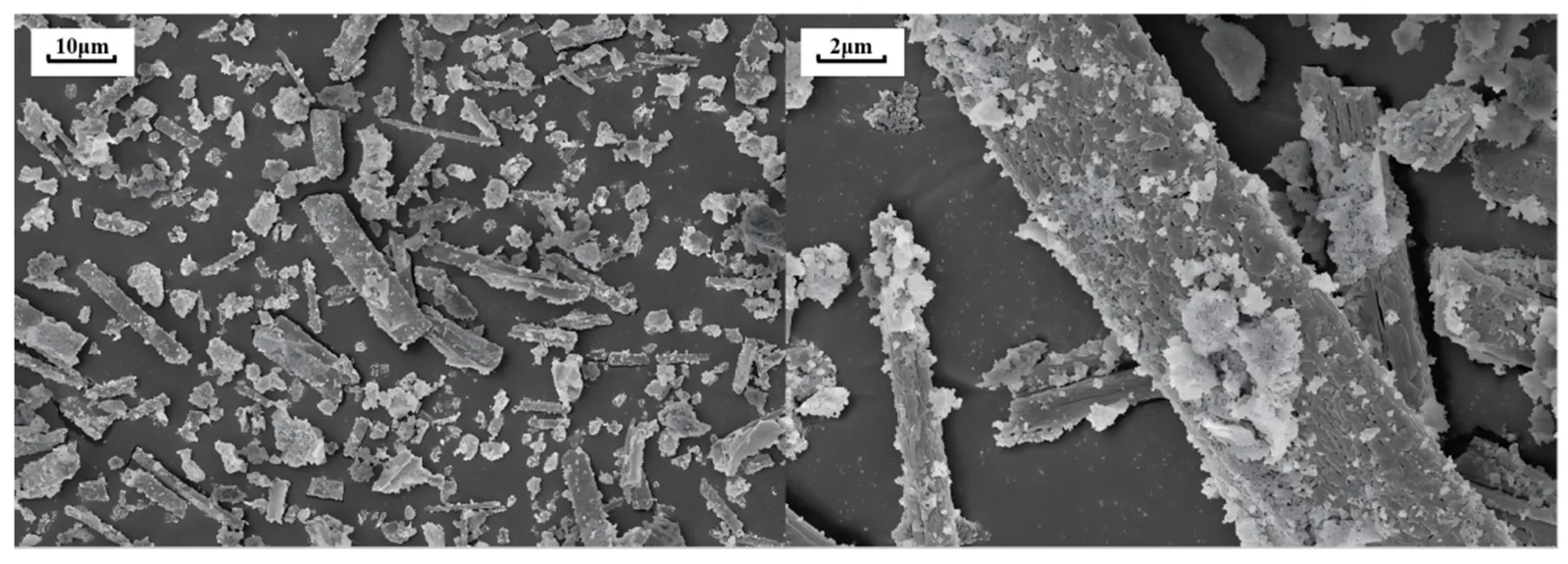
SEM image of the recovered precipitate.

Solid-phase characterization was then used to examine the conversion of magnesite-derived Mg^2+^ into crystalline precipitates. As shown in Figure 2, the raw magnesite particles showed irregular block-like morphologies under scanning electron microscopy (SEM), consistent with mechanically crushed mineral powders. After electrochemical reaction, the cathodic deposits (Figure 3) exhibited short prismatic and thick plate-like crystals, which are consistent with reported struvite morphologies [26,27,28,29]. Struvite, magnesium ammonium phosphate hexahydrate (MgNH_4_PO_4_·6H_2_O), commonly forms euhedral or platy crystals under alkaline conditions when Mg^2+^, NH_4_^+^ and phosphate species coexist. The morphological change from irregular mineral particles to ordered crystals indicates the formation of new crystalline precipitates during electrochemical activation.

The change in solid-phase composition further supported phosphorus enrichment in the recovered product. X-ray fluorescence spectroscopy (XRF) conventionally reports elemental compositions on an oxide-equivalent basis. As shown in Table 1, the raw magnesite had an MgO-equivalent content of 72.110 wt% and a P_2_O_5_-equivalent content of only 0.212 wt%. XRF analysis of the recovered product yielded P_2_O_5_- and MgO-equivalent contents of 47.323 and 26.234 wt%, respectively. These oxide-equivalent values do not indicate the presence of free P_2_O_5_ or MgO phases in the samples. Rather, the marked increase in P_2_O_5_-equivalent content indicates substantial phosphorus enrichment in the recovered product. Together with the XRD and SEM-EDS results, these data support the formation of phosphate-containing precipitates, including struvite.

**Figure 4:**
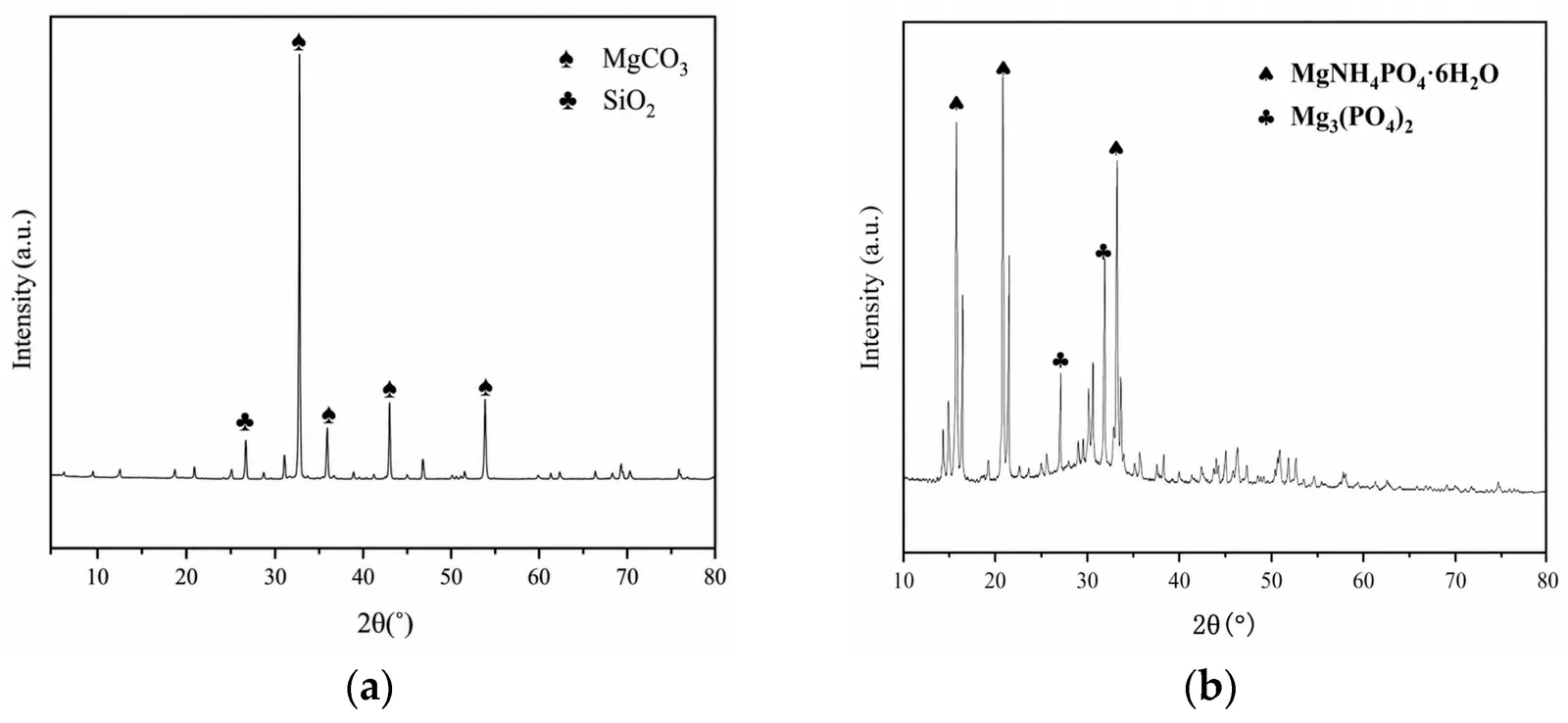
XRD pattern of raw magnesite (**a**) and pattern of the product (**b**).

X-ray diffraction (XRD) was used to identify the crystalline phases of the recovered product. As shown in Figure 4, the raw magnesite exhibited characteristic diffraction peaks of magnesium carbonate (MgCO_3_). After electrochemical reaction, the MgCO_3_ peaks disappeared, and the recovered product was dominated by diffraction peaks assigned to struvite (MgNH_4_PO_4_·6H_2_O, PDF#04-009-6297). Additional weaker peaks corresponding to Mg_3_(PO_4_)_2_ were also observed, indicating the coexistence of a non-target magnesium phosphate phase. These results support the formation of struvite-containing precipitates rather than a phase-pure struvite product.

### 2.2. Effect of Operating Parameters on Phosphorus Recovery and NH_4_^+^–N Apparent Removal Performance

The effects of current density, initial N:P molar ratio, initial phosphorus concentration and initial pH on PO_4_^3−^–P recovery, NH_4_^+^–N apparent removal, cathodic deposition and apparent MAP purity were examined. These experiments were used to define suitable operating conditions and to qualitatively interpret how Mg^2+^ release, cathodic alkalization, and ion availability influenced the development of conditions favorable for struvite nucleation and crystal growth.

#### 2.2.1. Effect of Current Density on NH_4_^+^–N Apparent Removal, PO_4_^3−^–P Recovery, Product Deposition Location and Apparent MAP Purity

**Figure 5:**
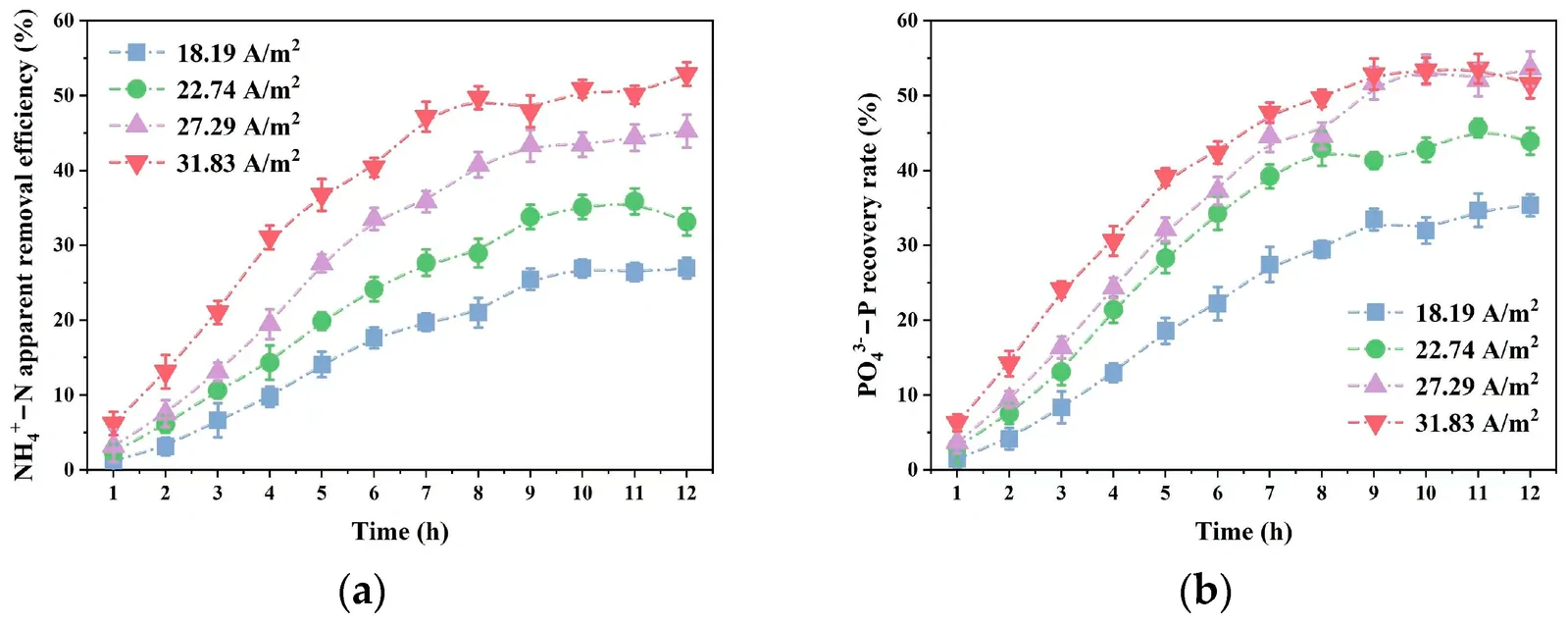
Effects of current density on (**a**) NH_4_^+^–N apparent removal efficiency and (**b**) PO_4_^3−^–P recovery efficiency.

Current density directly affected Mg^2+^ release from magnesite and OH^−^ generation at the cathode, thereby changing the local conditions for struvite precipitation. As shown in Figure 5, higher current density accelerated nutrient removal, but NH_4_^+^–N apparent removal and PO_4_^3−^–P recovery followed different trends. NH_4_^+^–N apparent removal increased from approximately 27% to 53% as the current density increased from 18.19 to 31.83 A/m^2^ after 12 h. This improvement was mainly associated with enhanced Mg^2+^ availability and stronger cathodic alkalization, which favored the enrichment of Mg^2+^, NH_4_^+^ and phosphate species near the cathode [13]. However, the higher NH_4_^+^–N apparent removal at elevated current density should be interpreted as apparent aqueous-phase removal rather than complete nitrogen recovery, since the intensified alkaline microenvironment may also shift part of the NH_4_^+^–N toward NH_3_ and cause nitrogen loss through volatilization or gas stripping.

Unlike NH_4_^+^–N apparent removal, PO_4_^3−^–P recovery showed a non-monotonic response to current density. The recovery efficiency increased from approximately 35% at 18.19 A/m^2^ to 53.58% at 27.29 A/m^2^, but no further improvement was observed at 31.83 A/m^2^. This suggests that phosphate recovery was not simply controlled by the amount of electrochemically generated Mg^2+^ or OH^−^. At a moderate current density, the observed phosphate recovery and product formation were consistent with the development of cathode-adjacent conditions favorable for heterogeneous struvite nucleation and subsequent crystal growth. When the current density was further increased, rapid alkalization and intensified gas evolution may have disturbed crystal attachment and favored the formation of non-target magnesium phases, such as Mg(OH)_2_ [30,31]. Based on the phosphate recovery performance and the need to limit excessive current input, 27.29 A/m^2^ was used in the subsequent experiments.

**Figure 6:**
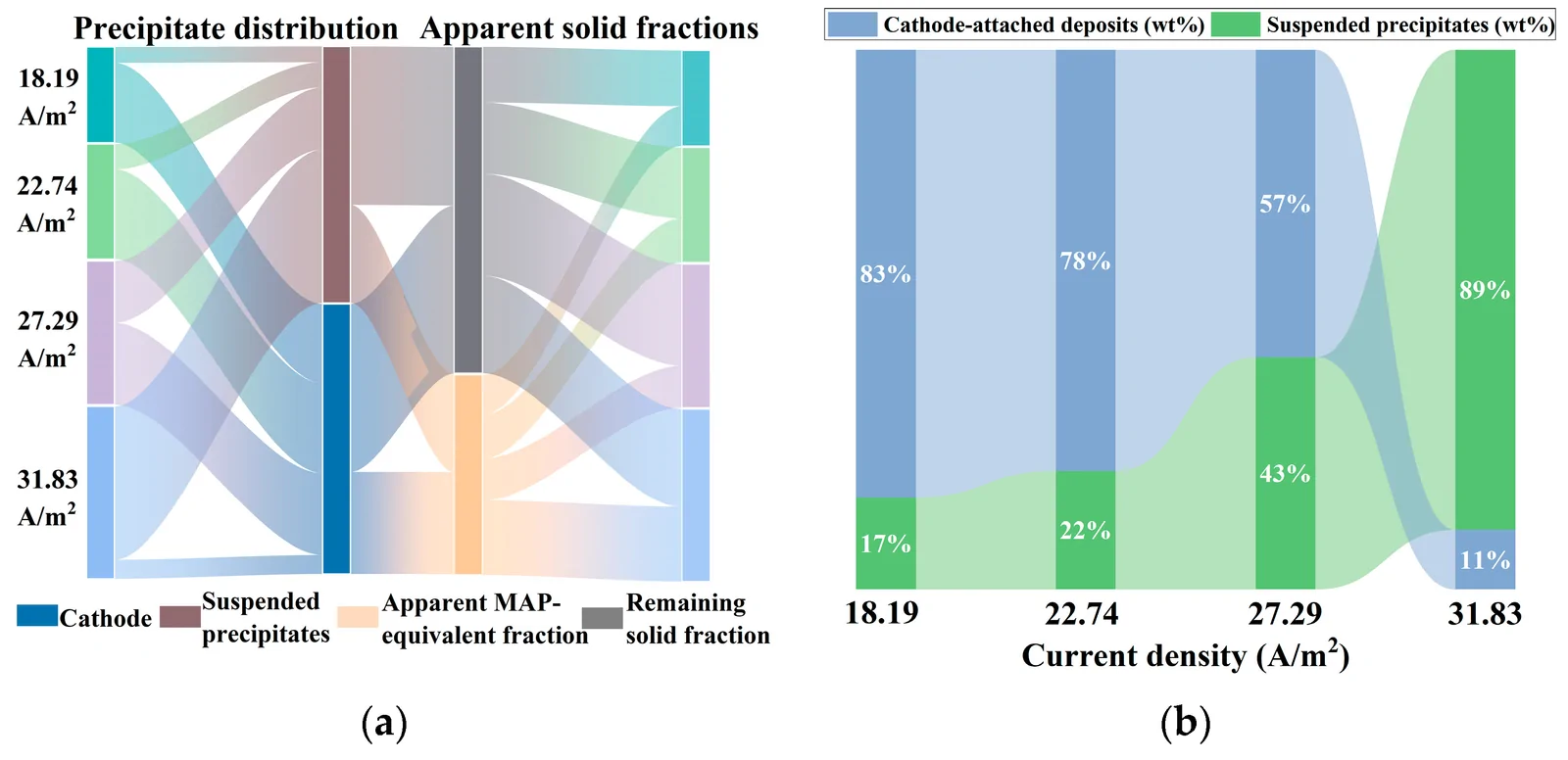
Effects of current density on (**a**) precipitate distribution and apparent MAP-equivalent and remaining solid fractions and (**b**) the relative mass fractions of cathode-attached deposits and suspended precipitates. The remaining solid fraction was calculated by difference.

Current density significantly affected the spatial distribution of the precipitates, as reflected by their partitioning between cathode-attached deposits and suspended precipitates in the bulk solution. As shown in Figure 6, the cathodic deposition fraction reached 83.11% at 18.19 A/m^2^ but decreased to 11.05% at 31.83 A/m^2^. This decrease was likely related to intensified hydrogen evolution, which disturbed the electrode–solution interface, weakened crystal attachment and promoted detachment of formed deposits. These results show that increasing current density improves Mg^2+^ release and alkalinity generation, but excessive current can reduce deposition stability through bubble disturbance and interfacial scouring.

#### 2.2.2. Effect of Initial N:P Molar Ratio on NH_4_^+^––N Apparent Removal and PO_4_^3−^–P Recovery

**Figure 7:**
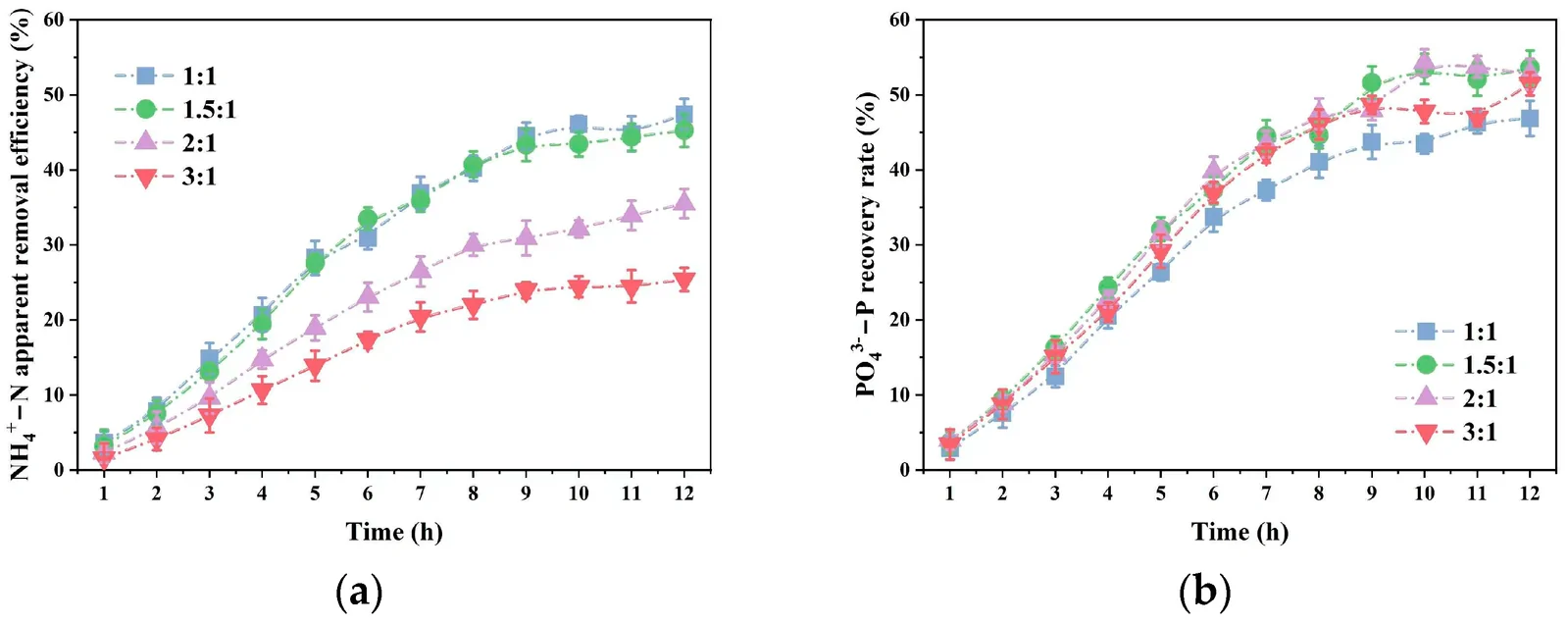
Effects of the N:P molar ratio on (**a**) NH_4_^+^–N apparent removal efficiency and (**b**) PO_4_^3−^–P recovery efficiency.

The initial N:P molar ratio markedly affected the ionic balance and nutrient conversion behavior during electrochemical struvite formation. As shown in Figure 7, all systems exhibited time-dependent increases in both NH_4_^+^–N apparent removal and PO_4_^3−^–P recovery, with rapid improvement during the initial reaction stage followed by a gradual plateau. Increasing the N:P ratio from 1:1 to 1.5:1 enhanced PO_4_^3−^–P recovery, which increased from approximately 47.0% to 53.5% at 12 h, while NH_4_^+^–N apparent removal remained at a comparable level. This result suggests that a moderate excess of ammonium helped maintain a favorable local ionic balance among Mg^2+^, NH_4_^+^, and phosphate species near the cathode, thereby promoting heterogeneous struvite nucleation [24,32].

However, further increasing the N:P ratio to 2:1 and 3:1 did not further improve phosphorus recovery, which stabilized at approximately 52–54%, whereas NH_4_^+^–N apparent removal decreased markedly to about 35.8% and 25.3%, respectively. This decline can be attributed to the increasing limitation of phosphate relative to ammonium under high N:P conditions, which restricted the stoichiometric incorporation of NH_4_^+^ into struvite. In addition, the local alkaline microenvironment near the cathode may have shifted part of the NH_4_^+^ toward NH_3_ volatilization, contributing to apparent nitrogen loss rather than recoverable struvite formation [13]. Therefore, the N:P ratio should be optimized by jointly considering PO_4_^3−^––P recovery, NH_4_^+^–N fate, and product quality, rather than by maximizing NH_4_^+^––N apparent removal alone. Based on these results, an N:P ratio of 1.5:1 was selected for subsequent experiments.

#### 2.2.3. Effect of Initial Phosphorus Concentration on NH_4_^+^–N Apparent Removal and PO_4_^3−^––P Recovery

**Figure 8:**
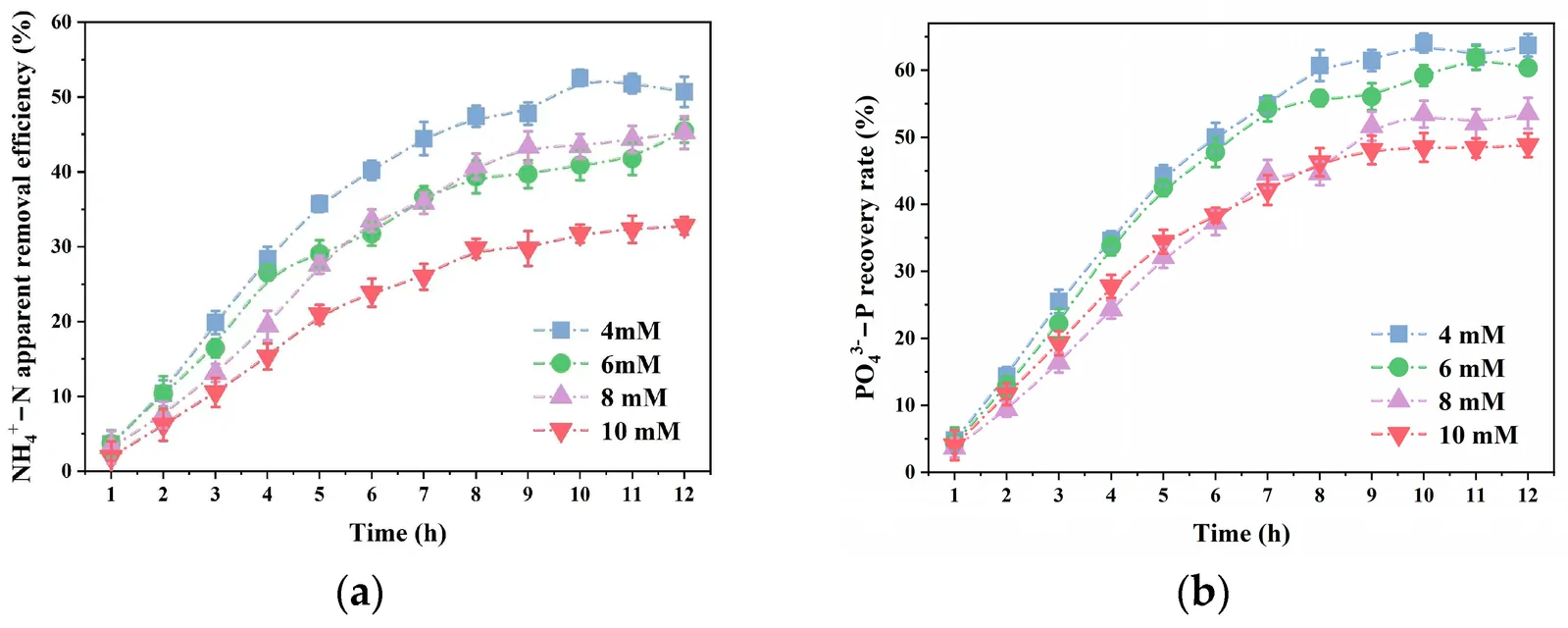
Effects of initial phosphorus concentration on (**a**) NH_4_^+^–N apparent removal efficiency and (**b**) PO_4_^3−^–P recovery efficiency.

The effect of initial phosphorus concentration was examined to identify a suitable loading range for electrochemically induced struvite recovery. As shown in Figure 8, both NH_4_^+^–N apparent removal and PO_4_^3−^––P recovery increased rapidly during the initial reaction stage and then gradually approached a plateau. Among the tested conditions, 4 mM exhibited the highest percentage removal performance, with NH_4_^+^––N apparent removal and PO_4_^3−^––P recovery reaching approximately 50% and 64% at 12 h, respectively. However, increasing the initial phosphorus concentration to 8 mM still maintained relatively high recovery performance, with a final NH_4_^+^–N apparent removal of approximately 45–46% and a mean final PO_4_^3−^–P recovery of 53.58%. More importantly, although the percentage recovery at 8 mM was lower than that at 4 mM, the corresponding phosphate concentration decrease was substantially greater, increasing from approximately 2.55 mM at 4 mM to 4.29 mM at 8 mM. This higher loading condition provided sufficient precipitate yield for subsequent product characterization while maintaining acceptable nutrient recovery efficiency.

Further increasing the initial phosphorus concentration to 10 mM did not provide a proportional improvement in recoverable product formation. Instead, NH_4_^+^–N apparent removal decreased markedly to approximately 32%, while PO_4_^3−^––P recovery declined to about 49%, suggesting that excessive phosphate loading may aggravate Mg^2+^ supply limitation, interfacial mass-transfer resistance and competition for cathodic crystallization sites. In addition, excessive phosphate may disturb the local Mg^2+^–NH_4_^+^–phosphate stoichiometric balance, thereby reducing the fraction of phosphate converted into recoverable struvite. Previous studies also reported that insufficient phosphate favors Mg(OH)_2_ formation, whereas appropriate phosphate loading promotes struvite crystallization through supersaturation control [30,33]. Therefore, the initial phosphorus concentration should be optimized not only according to percentage recovery but also by considering absolute nutrient recovery, solid product yield and downstream characterization requirements. Based on this trade-off, 8 mM was selected as the representative initial phosphorus concentration for subsequent experiments.

#### 2.2.4. Effect of Initial pH on NH_4_^+^–N Apparent Removal and PO_4_^3−^–P Recovery

**Figure 9:**
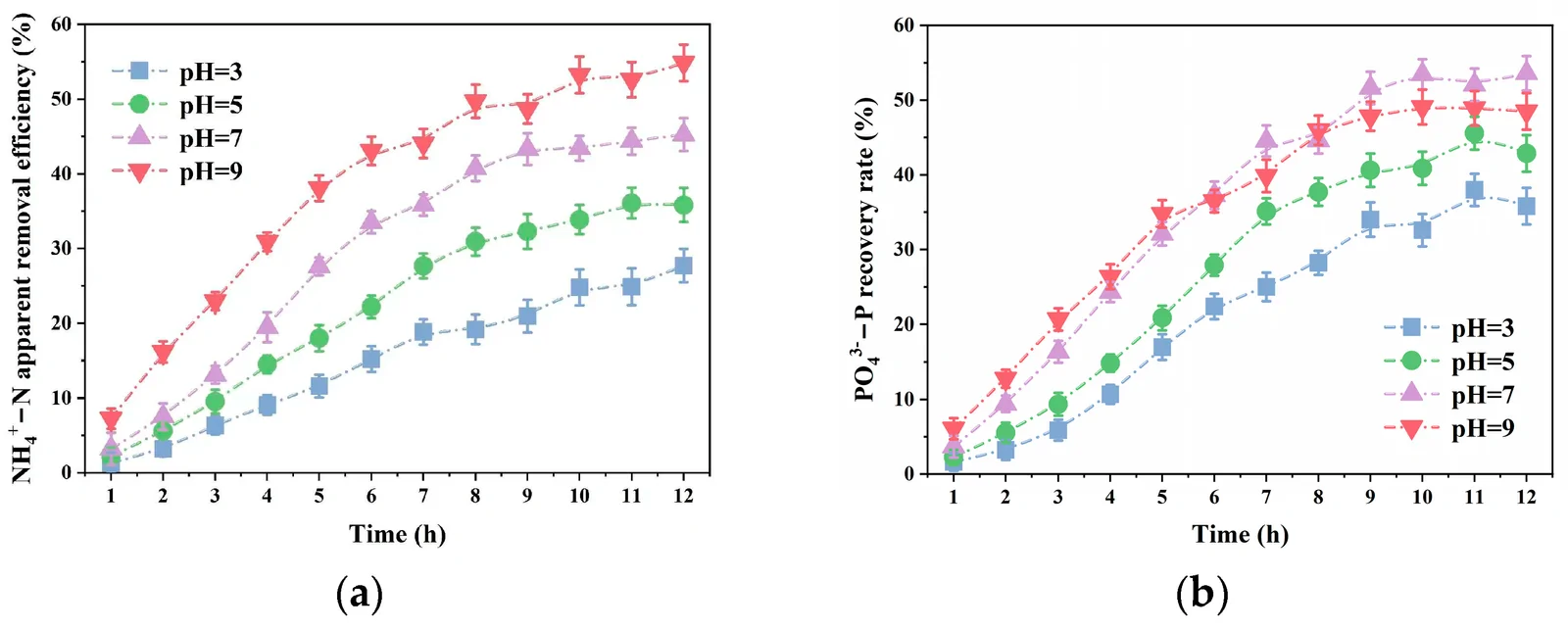
Effects of initial pH on (**a**) NH_4_^+^–N apparent removal efficiency and (**b**) PO_4_^3−^–P recovery efficiency.

Initial pH played a critical role in regulating struvite crystallization by affecting phosphate speciation, the NH_4_^+^/NH_3_ equilibrium, and the development of cathode-adjacent conditions favorable for precipitation. As shown in Figure 9, both NH_4_^+^–N apparent removal and PO_4_^3−^–P recovery increased with reaction time under all tested pH conditions, but their responses to initial pH were different. The NH_4_^+^–N apparent removal efficiency increased continuously as the initial pH increased from 3.0 to 9.0, reaching approximately 28%, 36%, 46% and 55% at pH 3.0, 5.0, 7.0 and 9.0 after 12 h, respectively. In contrast, PO_4_^3−^–P recovery increased from about 36% at pH 3.0 to 43% at pH 5.0 and reached the highest value of approximately 54% at pH 7.0, whereas a further increase to pH 9.0 resulted in a slight decrease to about 49%. This discrepancy indicates that NH_4_^+^–N apparent removal and PO_4_^3−^–P recovery were not governed by identical pathways, and that electrochemical phosphorus recovery depended strongly on the cathode-adjacent alkaline microenvironment rather than on bulk pH alone [13,34].

At low initial pH, cathodically generated OH^−^ was largely consumed by excess H^+^ before the cathode-adjacent region reached the alkaline range favorable for struvite nucleation, thereby delaying precipitation. As the initial pH increased to neutral conditions, phosphate species became more favorable for crystallization, and the cathodic alkaline microenvironment could more effectively induce heterogeneous struvite nucleation and growth. However, although pH 9.0 produced the highest NH_4_^+^–N apparent removal, this enhancement may partly originate from the shift of NH_4_^+^ toward NH_3_ under alkaline conditions, leading to apparent nitrogen loss rather than recoverable struvite formation [13]. Meanwhile, excessive alkalinity may promote Mg(OH)_2_ or other non-target magnesium phases, thereby reducing phosphate conversion into struvite. Cai et al. also noted that product composition in electrochemical struvite systems is jointly affected by phosphorus concentration and electrochemical conditions and may shift between Mg(OH)_2_ and MgNH_4_PO_4_ [30]. Therefore, precipitation behavior in this system was controlled by the combined effect of initial pH and cathodic alkalization. Considering PO_4_^3−^–P recovery, NH_4_^+^–N fate and by-product control, an initial pH of approximately 7.0 was more favorable for selective struvite crystallization.

### 2.3. Optimization of Operating Parameters by Response Surface Methodology

Response surface methodology with a Box–Behnken design was applied to evaluate four variables: initial pH (A), current density (B), initial phosphorus concentration (C) and initial N:P molar ratio (D). The fitted quadratic models were statistically significant (*p* < 0.0001), with R^2^ values above 0.97, indicating adequate agreement between the experimental and predicted responses within the tested range. The optimized conditions were initial pH 7.75, current density 28.21 A/m^2^, initial phosphorus concentration 6.41 mM and N:P molar ratio 1.42:1. Under these conditions, the predicted NH_4_^+^–N apparent removal, PO_4_^3−^–P recovery and apparent MAP purity were 53.01%, 58.68% and 63.36%, respectively. Triplicate validation experiments gave corresponding values of 52.26 ± 0.415%, 57.56 ± 0.757% and 62.56 ± 0.55%, with deviations below 5%, supporting the reliability of the RSM optimization.

**Figure 10:**
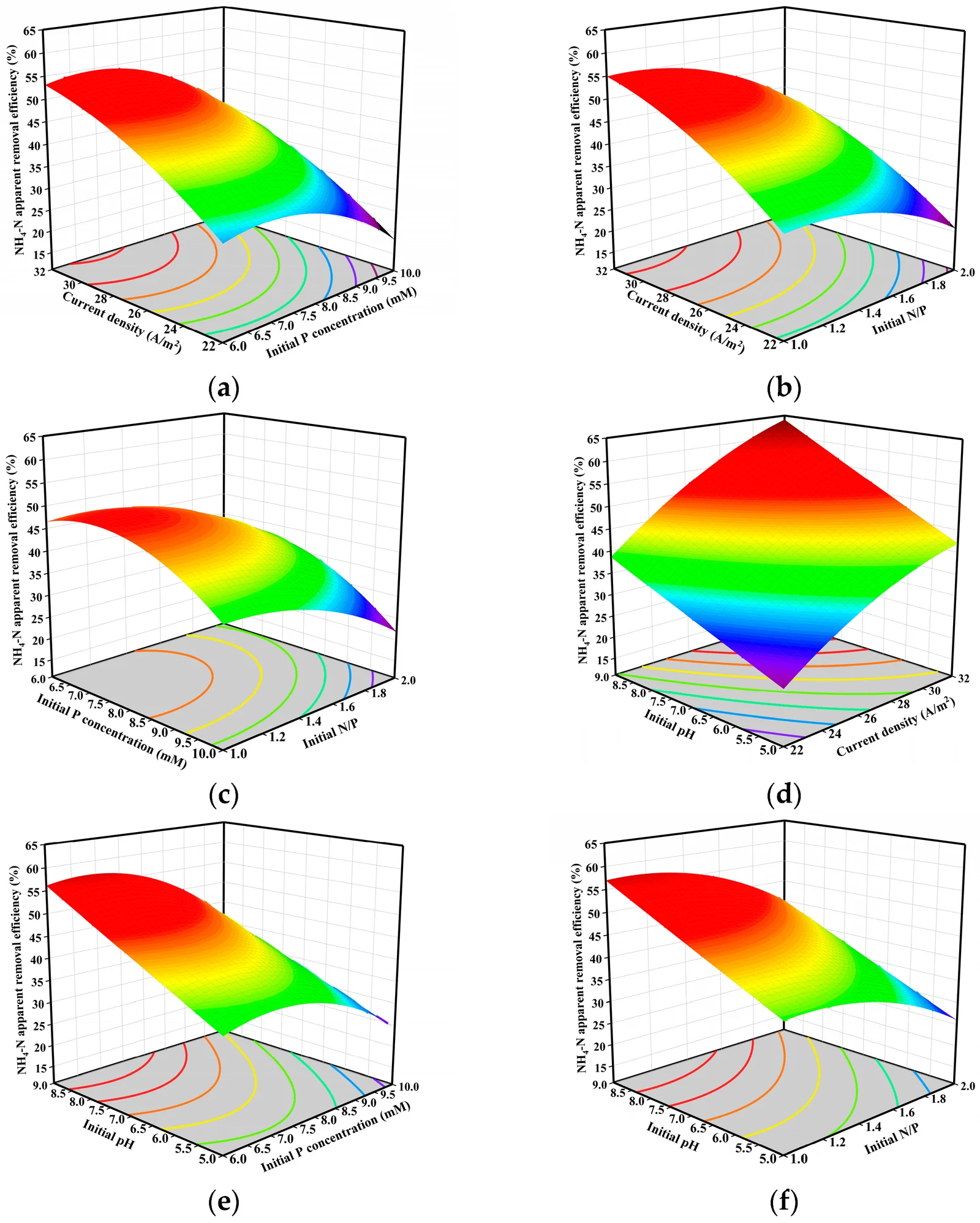
Response surface plots showing the pairwise interaction effects of (**a**) current density and initial P concentration, (**b**) current density and initial N:P molar ratio, (**c**) initial P concentration and initial N:P molar ratio, (**d**) initial pH and current density, (**e**) initial pH and initial P concentration, and (**f**) initial pH and initial N:P molar ratio on NH_4_^+^-N apparent removal efficiency.

For NH_4_^+^–N apparent removal, the response surfaces in Figure 10 show a clear dependence on current density and initial pH, while the effects of phosphorus concentration and N:P ratio were more closely related to substrate loading and stoichiometric balance. In the current density–phosphorus concentration plot, NH_4_^+^–N apparent removal increased with current density but tended to decrease when the initial phosphorus concentration was raised from 6 to 10 mM. This suggests that a higher current input promoted Mg^2+^ release and cathodic OH^−^ generation, whereas excessive phosphate loading may have exceeded the effective Mg^2+^ supply and interfacial crystallization capacity. A similar trend was observed for the current density–N:P interaction. Higher current density improved NH_4_^+^–N apparent removal, but increasing the initial N:P ratio did not further enhance the response, probably because the larger ammonium pool could not be proportionally converted into struvite under limited Mg^2+^ and phosphate availability.

The interaction involving initial pH was more pronounced. NH_4_^+^–N apparent removal increased as the initial pH shifted from weakly acidic to alkaline conditions, especially at higher current density. This behavior is reasonable because a higher initial pH reduced the amount of cathodically generated OH^−^ consumed by bulk acidity and helped the cathode-adjacent region reach the alkaline range required for precipitation more rapidly. However, the high NH_4_^+^–N apparent removal observed at elevated pH and current density should not be interpreted solely as nitrogen incorporation into MAP. Under strongly alkaline cathode-adjacent conditions, part of the NH_4_^+^ may be converted to NH_3_ and leave the system through volatilization or gas stripping. Therefore, aqueous NH_4_^+^–N apparent removal, nitrogen incorporation into MAP, and NH_3_ volatilization/stripping should be treated as distinct nitrogen pathways [35,36]. Because volatilized NH_3_ was not quantitatively captured in this study, the nitrogen mass balance could not be closed, and the reported NH_4_^+^–N response represents apparent aqueous-phase removal rather than complete nitrogen recovery. Future integration of quantitative off-gas capture or acid scrubbing would be required to close the nitrogen mass balance [36].

**Figure 11:**
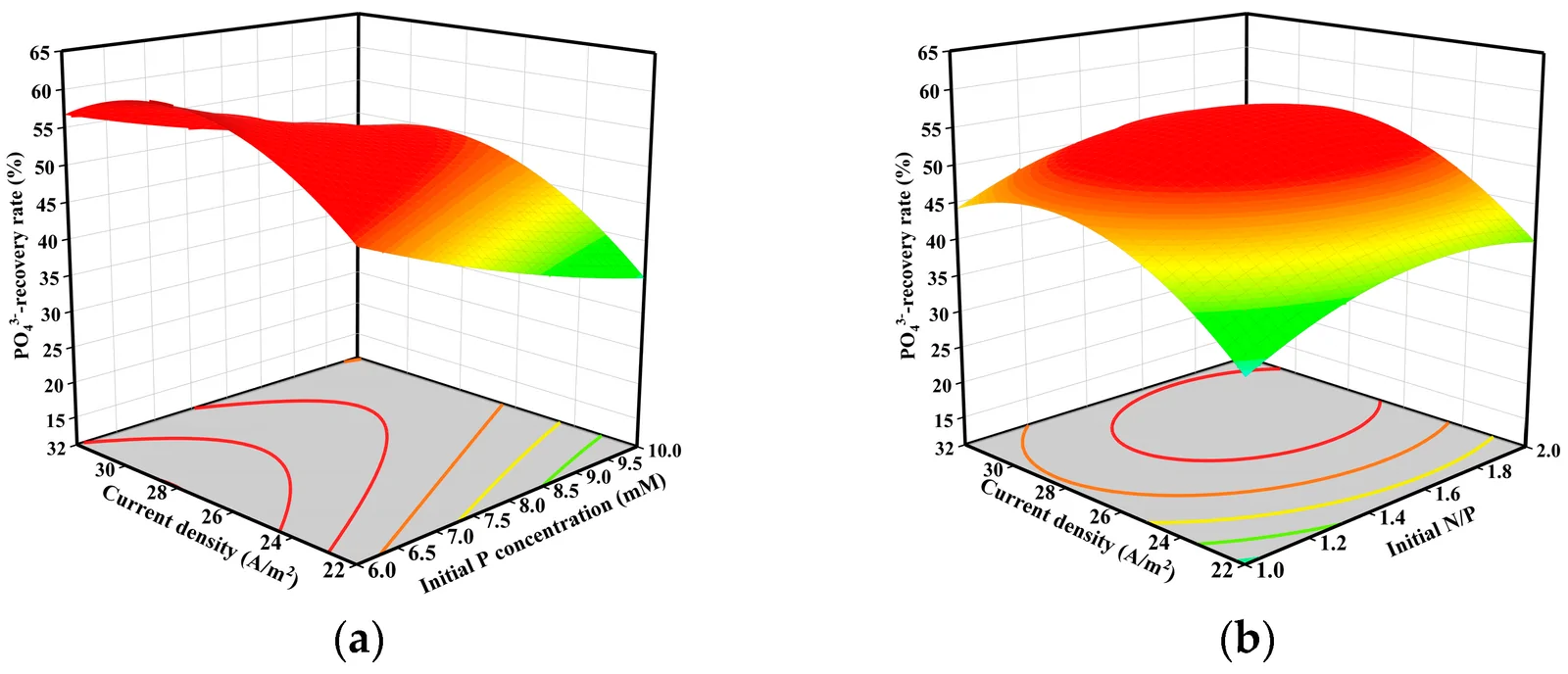

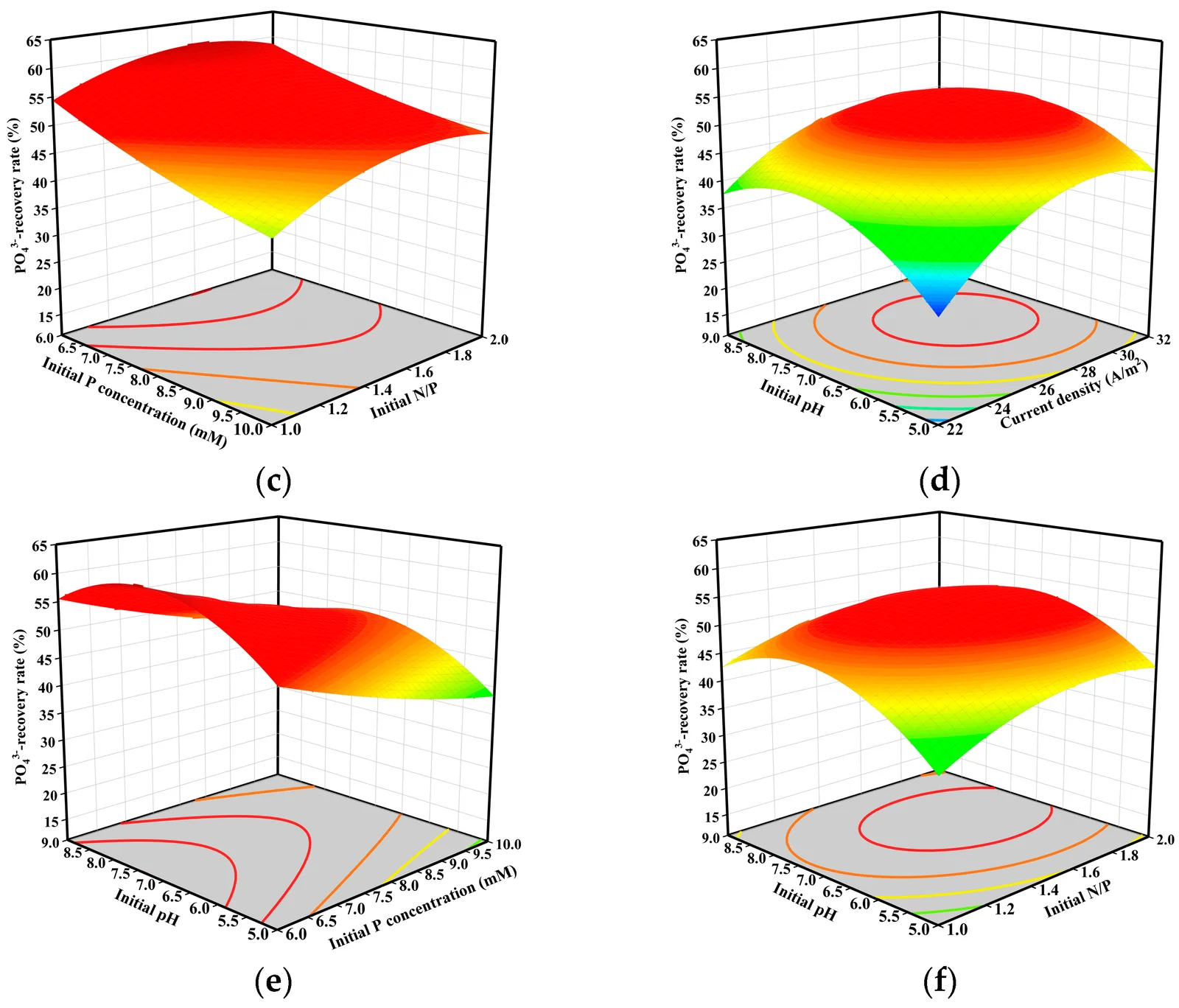
Response surface plots showing the pairwise interaction effects of (**a**) current density and initial P concentration, (**b**) current density and initial N:P molar ratio, (**c**) initial P concentration and initial N:P molar ratio, (**d**) initial pH and current density, (**e**) initial pH and initial P concentration, and (**f**) initial pH and initial N:P molar ratio on PO_4_^3−^-P recovery efficiency.

For PO_4_^3−^–P recovery, the response surfaces in Figure 11 indicate that phosphate recovery was governed by the combined effects of current density, initial phosphorus concentration, initial pH and N:P ratio. In the current density–phosphorus concentration surface, PO_4_^3−^–P recovery increased as current density rose from the lower range, but the improvement became limited at higher current input. Meanwhile, increasing the initial phosphorus concentration from 6 to 10 mM did not further enhance the percentage recovery. Instead, the recovery tended to decline at higher phosphorus loading, suggesting that phosphate conversion was no longer limited by phosphate availability but by Mg^2+^ release, local alkalization and interfacial crystallization capacity. A relatively favorable region appeared at moderate-to-high current density and low-to-medium phosphorus concentration, where Mg^2+^ supply and cathodic OH^−^ generation were better matched with phosphate precipitation.

The interactions involving pH and N:P ratio also showed clear nonlinear behavior. PO_4_^3−^–P recovery was higher under neutral to weakly alkaline conditions, especially around pH 7–8, while both lower pH and excessive alkalinity were less favorable. At low pH, part of the cathodically generated OH^−^ would be consumed before the cathode-adjacent region reached conditions favorable for struvite nucleation. At high pH, non-target magnesium phases or surface scaling may become more competitive, which could reduce the selectivity toward struvite. The N:P ratio showed a moderate optimum rather than a simple positive effect. An insufficient NH_4_^+^ supply limited MAP formation, whereas excessive NH_4_^+^ did not further improve phosphate recovery once Mg^2+^ and phosphate conversion became limiting. These trends are consistent with previous electrochemical phosphorus recovery studies showing that current density, solution pH, initial phosphorus concentration and electrode surface state jointly regulate phosphate precipitation through nonlinear interactions [31]. Overall, PO_4_^3−^–P recovery in this system was favored by a balanced condition, rather than by maximizing any single factor. A moderate current density, neutral to weakly alkaline pH, suitable N:P ratio and controlled phosphorus loading were more beneficial for balancing Mg^2+^ availability, nutrient stoichiometry, and cathodic alkalization, thereby supporting stable struvite deposition.

**Figure 12:**
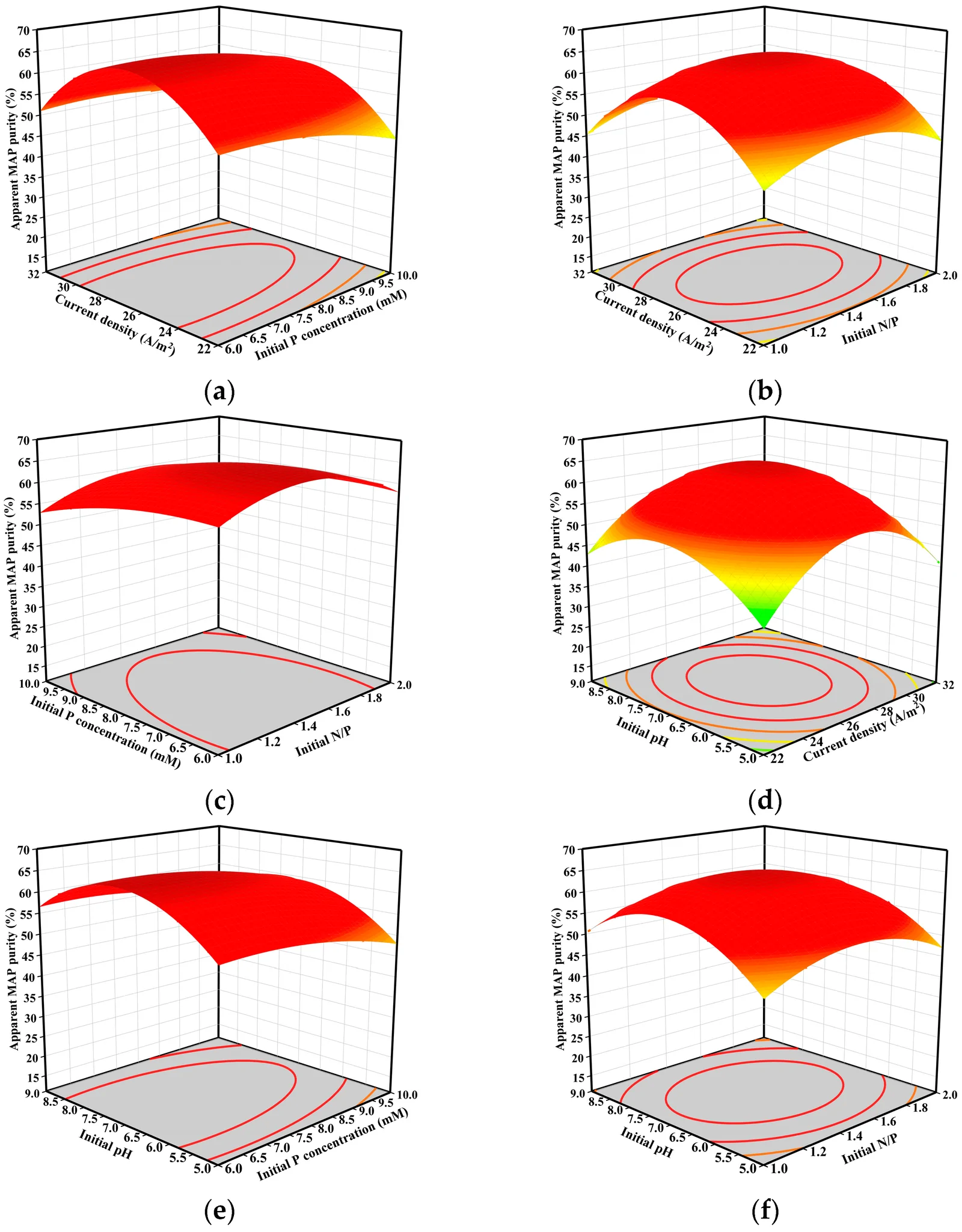
Response surface plots showing the pairwise interaction effects of (**a**) current density and initial P concentration, (**b**) current density and initial N:P molar ratio, (**c**) initial P concentration and initial N:P molar ratio, (**d**) initial pH and current density, (**e**) initial pH and initial P concentration, and (**f**) initial pH and initial N:P molar ratio on apparent MAP purity.

The apparent purity of MAP was also strongly affected by the interaction among operating factors, and the conditions favoring high purity were not identical to those giving the highest NH_4_^+^–N apparent removal. As shown in Figure 12, higher apparent MAP purity was mainly obtained under moderate current density and near-neutral initial pH. The response surface for current density and pH showed a clear dome-shaped profile, with the favorable region located around 27 A/m^2^ and pH 7. Under this condition, cathodic OH^−^ generation was sufficient to induce struvite nucleation, while excessive alkalization and gas disturbance were avoided. When the current density was further increased, stronger hydrogen evolution and rapid interfacial pH elevation may have disturbed crystal attachment and promoted the formation of non-target magnesium phases, leading to a decrease in apparent MAP purity.

Initial phosphorus concentration and N:P ratio also influenced product selectivity. The region with higher apparent MAP purity appeared at moderate phosphorus loading, roughly around 7–8 mM, while excessive phosphorus concentration tended to lower the purity. This may be related to a mismatch between phosphate supply, Mg^2+^ release and the available cathodic growth sites. Similarly, an appropriate N:P ratio favored MAP formation, whereas either insufficient or excessive ammonium supply reduced the apparent purity. These results suggest that MAP purity was governed by the balance among local alkalinity, Mg^2+^ availability, ion diffusion and crystal growth, rather than by simply increasing reaction intensity or nutrient loading [33]. Overall, a moderate current density, near-neutral pH, suitable phosphorus concentration and balanced N:P ratio were more favorable for obtaining a product with a relatively high apparent MAP fraction while maintaining nutrient recovery performance.

The response denoted as apparent MAP purity was calculated from the ammoniacal nitrogen released after acid dissolution of the washed deposits. It therefore represents an operational MAP-equivalent fraction used to compare product selectivity among the tested conditions, rather than an absolute quantitative phase composition. Accordingly, the RSM results describe relative changes in this operational response and should be interpreted together with the XRD, SEM-EDS, and FTIR characterization results.

### 2.4. Characterization of Recovered Products and Mechanistic Discussion

#### 2.4.1. Characterization of Recovered Products

**Figure 13:**
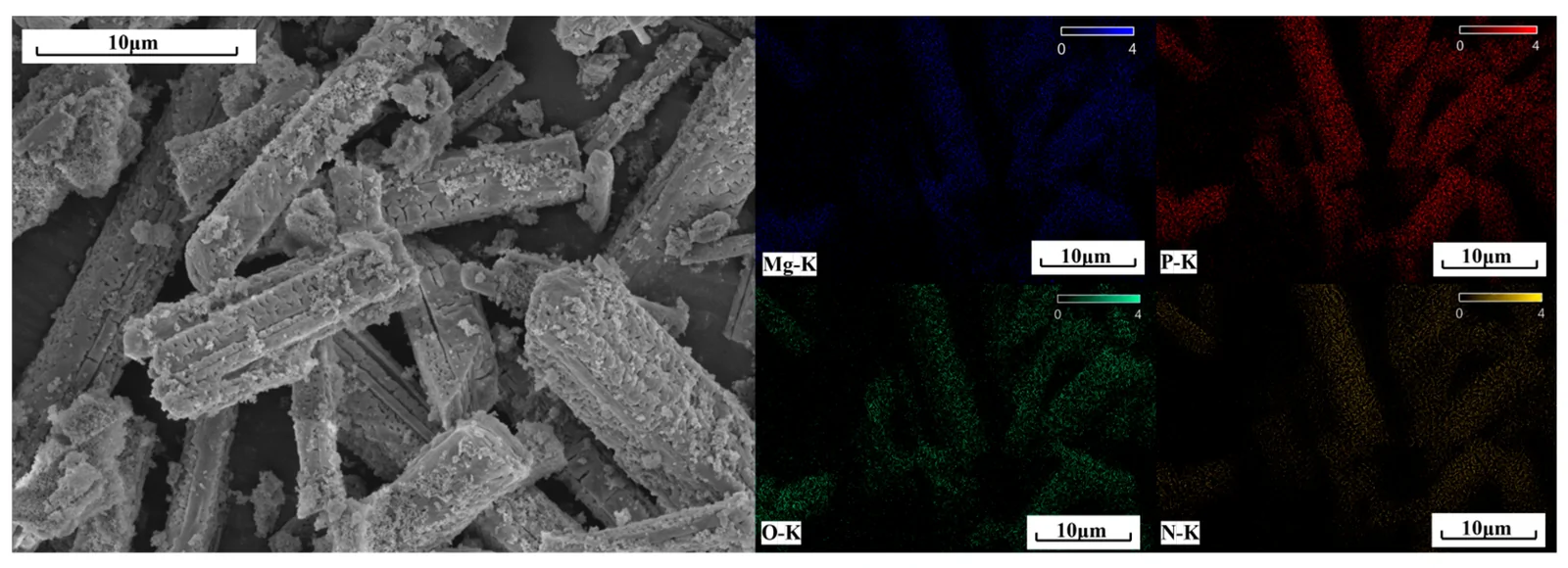
SEM image and EDS elemental mapping of recovered products obtained under the optimized conditions.

The products obtained under the optimized conditions, namely initial pH 7.75, current density 28.21 A/m^2^, initial phosphorus concentration 6.41 mM and N:P molar ratio 1.42:1, were characterized by X-ray diffraction (XRD), scanning electron microscopy (SEM) and energy-dispersive X-ray spectroscopy (EDS). As shown in Figure 13, the recovered products showed square-prismatic, short-prismatic and wedge-like crystals, which are consistent with reported struvite morphologies [27,33]. EDS mapping further showed the coexistence of Mg, N, P and O on the particle surfaces, supporting the formation of Mg–N–P-containing precipitates.

**Figure 14:**
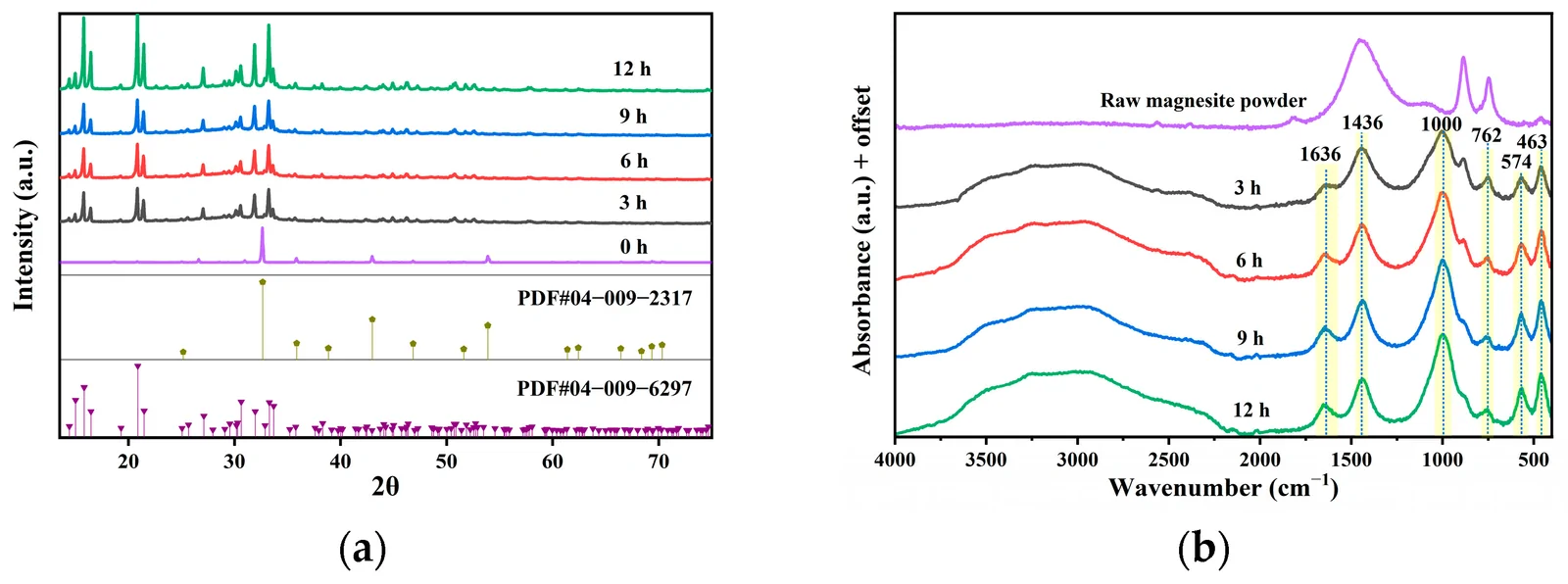
Time−dependent characterization of solid products during electrochemical magnesite activation: (**a**) XRD patterns and (**b**) FTIR spectra.

To follow the mineral transformation during electrolysis, solids collected at different reaction times were analyzed by XRD and Fourier-transform infrared spectroscopy (FTIR). As shown in Figure 14a, the raw material at 0 h showed characteristic diffraction peaks of MgCO_3_ (PDF#04-009-2317). After 3 h of reaction, diffraction peaks assigned to struvite (PDF#04-009-6297) appeared, and the phosphorus recovery efficiency reached 22.34%, indicating early formation of struvite while residual magnesite was still present. At 9 h, phosphorus recovery increased to 55.69%, the struvite peaks became stronger, and the magnesite peaks were barely observed, suggesting that struvite became the principal crystalline phase detected by XRD and that the crystalline phase assemblage changed only slightly thereafter. This qualitative phase identification does not provide the absolute mass fraction of struvite in the recovered solid.

FTIR spectra of raw magnesite powder and the recovered solids collected after electrochemical treatment are presented in Figure 14b. The broad absorption band in the 3800–2200 cm^−1^ region was attributed to overlapping O-H stretching vibrations of crystal water and N-H stretching vibrations of NH_4_^+^ associated with hydrated phosphate precipitates [37]. The band at approximately 1636 cm^−1^ was assigned to the H-O-H bending vibration of crystal water. The absorption band near 1436 cm^−1^ was mainly associated with NH_4_^+^ bending vibration, with possible contribution from carbonate vibrations of residual magnesite [38,39]. The characteristic bands at approximately 1000, 574, and 463 cm^−1^ were related to PO_4_^3−^ stretching and bending vibrations, which are characteristic of struvite and other magnesium phosphate phases [40,41]. The band around 762 cm^−1^ was associated with hydrogen-bond interactions involving crystal water molecules. Compared with raw magnesite, the emergence of phosphate-related bands in the recovered solids supports the formation of struvite-containing precipitates, suggesting that Mg^2+^ released from electrochemically activated magnesite contributed to phosphate recovery. These FTIR features were consistent with the XRD results and support the formation of struvite-containing products.

#### 2.4.2. Mechanistic Discussion

**Figure 15:**
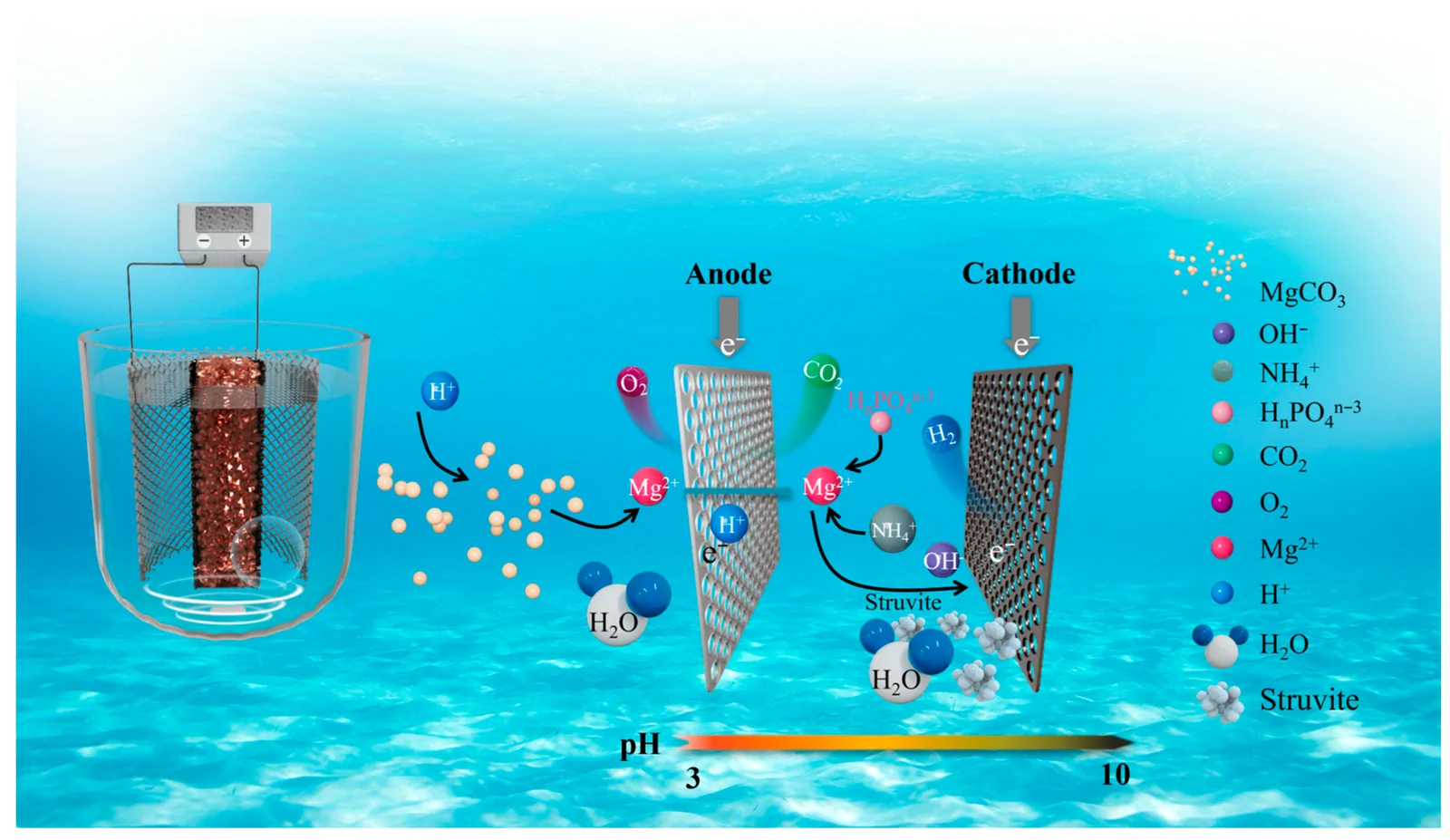
Schematic illustration of electrochemically induced MAP crystallization.

The electrochemical behavior and product characterization suggest that phosphorus recovery in this system occurred through a spatially coupled electrochemical–crystallization process rather than simple bulk precipitation driven by external magnesium addition. At the anode, water oxidation generated H^+^ and created an acidic microregion that promoted magnesite dissolution and Mg^2+^ release. At the cathode, water reduction produced OH^−^ and established an alkaline microenvironment favorable for phosphate precipitation. Under the combined effects of electric-field-driven migration, diffusion, and convection, Mg^2+^, NH_4_^+^, and phosphate species could be transported toward the cathode-adjacent region. The resulting interfacial environment was qualitatively inferred to favor heterogeneous struvite nucleation and crystal growth (Figure 15). Although the pH of the solution adjacent to the cathode was monitored, the microscale interfacial pH and local concentrations of Mg^2+^, NH_4_^+^, and phosphate species within the cathodic diffusion layer were not directly resolved, and the local saturation index with respect to struvite was not calculated. Accordingly, the proposed local supersaturation should be regarded as a mechanistic inference rather than a directly measured parameter. Part of the NH_4_^+^ could also be converted to NH_3_·H_2_O/NH_3_ under alkaline conditions and leave the system through volatilization. Thus, the observed NH_4_^+^–N apparent removal represents the combined contribution of nitrogen incorporation into MAP and ammonia volatilization, rather than complete nitrogen recovery.

This interpretation is consistent with previous electrochemical precipitation studies showing that local pH gradients and ion transport can create electrode-adjacent conditions favorable for phosphate mineral formation and influence the deposition location [13,31,42]. In the present system, continuous H^+^ production at the anode promoted acid dissolution of MgCO_3_ and sustained Mg^2+^ release from magnesite particles. Simultaneously, OH^−^ generated at the cathode was expected to increase the pH within the cathodic diffusion layer relative to the bulk solution, thereby favoring precipitation near the electrode. The cathode therefore likely served as both the source of local alkalinity and a principal region for interfacial nucleation and deposition.

Within this cathode-adjacent alkaline region, Mg^2+^ released from magnesite, together with NH_4_^+^ and HPO_4_^2−^/PO_4_^3−^ from wastewater, could become available near the cathode interface. Considering the operating pH range of this system, HPO_4_^2−^ is likely to be the more representative reactive phosphate species [43]. Therefore, the reaction Mg^2+^ + NH_4_^+^ + HPO_4_^2−^ + 6H_2_O → MgNH_4_PO_4_·6H_2_O(s) + H^+^ may better describe the main precipitation pathway, whereas the PO_4_^3−^-based reaction should be regarded as a simplified overall expression under more alkaline conditions. Time-resolved studies have shown that struvite can nucleate and grow directly from solution in a pure Mg–NH_4_–PO_4_ system, whereas transient amorphous phosphate phases were observed under specific Co-containing conditions [44]. Because transient intermediates were not directly observed in the present study, no specific amorphous-precursor-mediated crystallization pathway is assigned here. The mechanistic interpretation is therefore limited to the measured Mg^2+^ release and cathode-adjacent alkalization, together with the inferred roles of ion transport and heterogeneous struvite nucleation and growth.

Excessive local alkalization may accelerate precipitation, but it may also shift the NH_4_^+^/NH_3_ equilibrium toward NH_3_ volatilization and promote the formation of Mg(OH)_2_ or amorphous magnesium phosphate side phases. These competing pathways can reduce apparent MAP purity and process selectivity. Therefore, effective phosphorus recovery in this system depends on matching anodic Mg^2+^ release, cathodic OH^−^ generation, ion transport and interfacial nucleation, rather than simply increasing the reaction intensity.

2H_2_O → O_2_ + 4H^+^ + 4e^−^ (1)

2H_2_O + 2e^−^ → H_2_ + 2OH^−^ (2)

MgCO_3_ + 2H^+^ → Mg^2+^ + CO_2_ + H_2_O (3)

Mg^2+^ + NH_4_^+^ + HPO_4_^2−^ + 6H_2_O → MgNH_4_PO_4_·6H_2_O(s) + H^+^ (4)

Mg^2+^ + NH_4_^+^ + PO_4_^3−^ + 6H_2_O → MgNH_4_PO_4_·6H_2_O(s) (5)

NH_4_^+^ + OH^−^ ⇌ NH_3_·H_2_O ⇌ NH_3_(g) + H_2_O (6)

## 3. Materials and Methods

### 3.1. Materials and Chemicals

Natural magnesite was obtained from a mining plant in Shanxi Province, China. The raw ore was crushed with a jaw crusher, ground in a planetary ball mill and sieved using standard sieves. The magnesite fraction with a particle size range of 1–2 mm was selected for subsequent experiments, dried at 105 °C for 24 h in a forced-air drying oven (DH-101, Tianjin Zhonghuan, Tianjin, China), and stored in a desiccator before use.

Ammonium chloride (NH_4_Cl), disodium hydrogen phosphate (Na_2_HPO_4_), sodium sulfate (Na_2_SO_4_), hydrochloric acid (HCl) and sodium hydroxide (NaOH) were of analytical grade and purchased from Shanghai Aladdin Reagent Co., Ltd. (Shanghai, China). Deionized water was used in all experiments.

### 3.2. Experimental Setup

Electrochemical experiments were performed in a custom-made cylindrical plexiglass electrolytic cell with an internal diameter of 10 cm, an internal height of 12 cm, and a working volume of 0.800 L. A Ru-Ir/Ti-coated titanium mesh served as the inner cylindrical anode, with a diameter of 2 cm, an immersed height of 10 cm, and an effective immersed geometric area of 65.97 cm^2^ (6.597 × 10^−3^ m^2^). The 316 L stainless-steel perforated-mesh cathode was configured as the outer cylindrical electrode, with a diameter of 8 cm, a total height of 12 cm, and an effective immersed height of 10 cm. The two cylindrical electrodes were arranged concentrically, resulting in a radial inter-electrode distance of 3 cm.

The electrodes were connected to a regulated DC power supply (DH1766-1, Beijing Dahua, Beijing, China) operated under constant-current mode. All current densities reported in this study were normalized to the effective immersed geometric area of the anode. Accordingly, anodic current densities of 13.64, 18.19, 22.74, 27.29, and 31.83 A/m^2^ corresponded to applied currents of 0.090, 0.120, 0.150, 0.180, and 0.210 A, respectively. During electrolysis, the suspension was stirred at 200 rpm using a magnetic stirrer (SH-5, Beijing Jinbeide, Beijing, China) to maintain mixing.

### 3.3. Experimental Procedures

#### 3.3.1. Feasibility Tests

Electrochemical activation of magnesite was first examined in the absence of added nitrogen and phosphorus. Specifically, 800 mL of deionized water containing 0.20 mol/L Na_2_SO_4_ as the supporting electrolyte and 40.0 g of pretreated, oven-dried magnesite particles with a particle size of 1–2 mm were added to the reactor. The magnesite dosage corresponded to a solid loading of 50.0 g/L. Electrolysis was conducted at nominal anodic current densities of 13.64, 18.19, 22.74, 27.29, and 31.83 A/m^2^, corresponding to applied currents of 0.090, 0.120, 0.150, 0.180, and 0.210 A, respectively. Samples were collected every 30 min, filtered through 0.45 μm membranes, and analyzed for dissolved Mg^2+^, Ca^2+^, and pH to evaluate mineral dissolution and alkalinity evolution.

#### 3.3.2. Batch Recovery Experiments

Synthetic nitrogen- and phosphorus-containing wastewater was prepared by dissolving NH_4_Cl and Na_2_HPO_4_ in deionized water containing 0.20 mol/L Na_2_SO_4_. In each batch experiment, 40.0 g of pretreated, oven-dried magnesite particles were added to 800 mL of synthetic wastewater, corresponding to a magnesite loading of 50.0 g/L. The same magnesite dosage was used in all single-factor experiments, Box–Behnken design runs, and validation experiments. The suspension was reacted at 25 ± 1 °C under continuous stirring at 200 rpm. The effects of anodic current density, initial N:P molar ratio, initial phosphorus concentration and initial pH on PO_4_^3−^–P recovery, NH_4_^+^–N apparent removal and product deposition were investigated. Current density was tested at 18.19–31.83 A/m^2^ under an initial PO_4_^3−^–P concentration of 8 mM, N:P ratio of 1.5:1 and pH 7.0. The N:P ratio was varied from 1:1 to 3:1 at 27.29 A/m^2^, 8 mM PO_4_^3−^–P and pH 7.0. The initial PO_4_^3−^–P concentration was varied from 4 to 10 mM at 27.29 A/m^2^, N:P = 1.5:1 and pH 7.0. The initial pH was adjusted to 3.0, 5.0, 7.0 and 9.0 at 27.29 A/m^2^, N:P = 1.5:1 and 8 mM PO_4_^3−^–P. Unless otherwise specified, all experiments were conducted at 200 rpm for 12 h. Samples were collected at predetermined intervals, filtered and analyzed for residual NH_4_^+^–N, PO_4_^3−^–P and Mg^2+^. After reaction, cathodic deposits and suspended precipitates were collected separately, washed three times with deionized water and dried at 40 °C for 24 h before characterization.

#### 3.3.3. Response Surface Methodology Optimization

Response surface methodology was performed using Design-Expert 12.0 with a Box–Behnken design to evaluate factor interactions and optimize operating conditions. A four-factor, three-level design was used. The independent variables were initial pH (A), current density (B, A/m^2^), initial phosphorus concentration (C, mM) and initial N:P molar ratio (D). The magnesite dosage was fixed at 40.0 g per 0.800 L of synthetic wastewater, corresponding to 50.0 g/L, in all Box–Behnken design runs and validation experiments. The reaction volume, stirring rate, temperature, and reaction time were maintained at 0.800 L, 200 rpm, 25 ± 1 °C, and 12 h, respectively. NH_4_^+^–N apparent removal efficiency, PO_4_^3−^–P recovery efficiency and apparent MAP purity were selected as the response variables. The coded levels of the independent variables are listed in Table 2.

### 3.4. Analytical and Characterization Methods

#### 3.4.1. Water Quality Analysis

NH_4_^+^–N was determined by Nessler’s reagent spectrophotometry according to HJ 535-2009, and PO_4_^3−^–P was measured by the molybdenum blue–ascorbic acid spectrophotometric method according to GB/T 11893-1989. Both measurements were conducted using a UV–Vis spectrophotometer (T6 New Century, Beijing Purkinje General Instrument Co., Ltd., Beijing, China). Mg and Ca concentrations were determined by inductively coupled plasma optical emission spectrometry (ICP-OES; ICPE-9820, Shimadzu, Japan).

#### 3.4.2. Product Characterization

The crystalline phases of dried precipitates were analyzed by X-ray diffraction (XRD; D8 Advance, Bruker, Karlsruhe, Germany) with Cu Kα radiation (λ = 0.15406 nm) over 10–80° at 8° min^−1^, 40 kV and 40 mA. Major elemental compositions were measured by X-ray fluorescence spectroscopy (XRF; EDX-7000, Shimadzu, Kyoto, Japan). Product morphology and crystal size were examined by scanning electron microscopy (SEM; SU8010, Hitachi, Tokyo, Japan) at 15 kV, and local elemental composition was obtained using SEM-coupled energy-dispersive X-ray spectroscopy (EDS). Functional groups and bond vibrations of the raw magnesite and dried precipitates were analyzed by Fourier-transform infrared spectroscopy (FTIR; Nicolet iS10, Thermo Fisher Scientific, Waltham, MA, USA) over 4000–400 cm^−1^ at a resolution of 4 cm^−1^ using KBr pellets prepared at a sample-to-KBr mass ratio of 1:100 and pressed under a load of 10 t for 60 s.

#### 3.4.3. Evaluation of Nutrient Removal, Cathodic Deposition, and Apparent MAP Purity

(1) NH_4_^+^–N apparent removal efficiency and PO_4_^3−^–P recovery efficiency

NH_4_^+^–N apparent removal efficiency and PO_4_^3−^–P recovery efficiency were calculated using Equations (7) and (8), respectively:

R_N_ (%) = [(C_N0_ − C_N_)/C_N0_] × 100 (7)

R_P_ (%) = [(C_P0_ − C_P_)/C_P0_] × 100 (8)

where R_N_ is the NH_4_^+^–N apparent removal efficiency (%), R_P_ is the PO_4_^3−^–P recovery efficiency (%), C_N0_ is the initial NH_4_^+^–N concentration (mg/L), C_N_ is the NH_4_^+^–N concentration after reaction (mg/L), C_P0_ is the initial PO_4_^3−^–P concentration (mg/L), and C_P_ is the PO_4_^3−^–P concentration after reaction (mg/L).

(2) Fraction of recovered solids deposited on the cathode

The amount of cathodic deposition and its fraction relative to the total precipitate were calculated as follows:

W_d_ = m_4_ − m_1_ (9)

R_d_ = (m_4_ − m_1_)/[(m_4_ − m_1_) + (m_3_ − m_2_)] (10)

where W_d_ is the mass of cathodic deposits (mg), R_d_ is the fraction of precipitates deposited on the cathode, m_1_ is the mass of the cathode before reaction (mg), m_2_ is the mass of the clean filter membrane before filtration (mg), m_3_ is the combined dry mass of the collected precipitates and filter membrane after reaction (mg), and m_4_ is the dry mass of the cathode after reaction (mg).

(3) Apparent MAP purity

The dry mass of cathode-attached deposits was determined from the difference between the dry cathode masses before and after reaction. Solids from the bulk solution were collected by filtration, washed with deionized water, dried, and weighed. To determine the apparent MAP purity of the combined recovered deposits, the cathode bearing the deposits and the dried bulk solids were subjected to acid treatment together using 100 mL of 5% HCl to dissolve the recovered solids. This step completely releases the constituent ions (specifically ammonium) into the aqueous phase for subsequent quantification. The solution was then transferred to a volumetric flask and diluted to a final volume of V_1_. The NH_4_^+^–N concentration in the solution was measured as c_1_. The MAP concentration in the dissolved deposit and the apparent MAP purity were calculated using Equations (11) and (12):

C_struvite_ = (c_1_ × M_struvite_)/M_N_ (11)

P_d_ = [(C_struvite_ × V_1_/1000)/W_1_] × 100% (12)

where C_struvite_ is the MAP concentration in the dissolved deposit (mg/L), c_1_ is the NH_4_^+^–N concentration determined by Nessler’s reagent spectrophotometry (mg/L), M_struvite_ is the molar mass of MAP (245,000 mg/mol), M_N_ is the molar mass of ammoniacal nitrogen in MAP (14,000 mg/mol), V_1_ is the final volume of the dissolved deposit solution (mL), W_1_ is the total dry mass of the cathode-attached deposits and the solids recovered from the bulk solution (mg), excluding the mass of the cathode substrate, and P_d_ is the apparent MAP purity of the deposit (%).

The calculated value is reported as apparent MAP purity because it represents an NH_4_^+^–N-based MAP-equivalent fraction rather than an absolute quantitative phase fraction. The deposits were washed three times with deionized water before analysis to minimize soluble ammonium residues. Non-nitrogenous solids, including Mg(OH)_2_, magnesium phosphate phases, and residual mineral components, contribute to the total deposit mass W_1_ but not to the measured ammoniacal-nitrogen concentration c_1_, and therefore decrease rather than increase the calculated MAP-equivalent fraction. Nevertheless, the calculation assumes that the ammoniacal nitrogen released during acid dissolution was primarily associated with MAP. Possible contributions from residual adsorbed or occluded NH_4_^+^ and undetected amorphous nitrogen-bearing material cannot be completely excluded. Therefore, P_d_ should be interpreted together with the XRD, SEM-EDS, and FTIR results, and determination of the absolute product composition would require independent quantitative phase analysis and elemental mass balance.

## 4. Conclusions

This work investigated electrochemical activation of natural magnesite as an in situ Mg source for struvite-based phosphorus recovery from wastewater. The system avoided soluble magnesium-salt dosing and reduced the need for continuous external alkali addition during electrolysis. Instead, Mg^2+^ release, cathodic alkalinity generation and MAP precipitation were coupled in one reactor. Anodic acidification promoted Mg^2+^ release from magnesite, while cathodic alkalization created a local alkaline zone for MAP nucleation and growth.

Current density played an important role in nutrient recovery and product formation. A low current density limited Mg^2+^ release and OH^−^ generation. Excessive current input enhanced NH_4_^+^–N apparent removal, but it did not further improve PO_4_^3−^–P recovery. It may also intensify hydrogen evolution and disturb crystal attachment near the cathode. Therefore, moderate current input was more favorable for selective MAP formation. The N:P molar ratio, initial phosphorus concentration and initial pH also affected the process. Within the tested range, moderate phosphorus loading was preferred. Excessive phosphate input could reduce the percentage recovery because Mg^2+^ supply, mass transfer and cathodic growth sites became limiting.

The RSM results gave an optimized condition of initial pH 7.75, current density 28.21 A/m^2^, initial phosphorus concentration 6.41 mM and N:P molar ratio 1.42:1. Under this condition, NH_4_^+^–N apparent removal, PO_4_^3−^–P recovery and apparent MAP purity reached 52.26%, 57.56% and 62.56%, respectively. These values agreed well with the model predictions. The reported apparent MAP purity represents an NH_4_^+^–N-based operational estimate and should not be interpreted as an absolute quantitative phase fraction. XRD, SEM-EDS, and FTIR supported the presence of MAP-containing products, whereas independent quantitative phase analysis and elemental mass balance would be required to determine the absolute product composition. The observed results were consistent with a process involving magnesite dissolution, Mg^2+^ transport, cathodic OH^−^ generation, and the development of cathode-adjacent conditions favorable for heterogeneous MAP crystallization. Because microscale ion concentrations and the local supersaturation index were not directly determined, this interfacial interpretation remains qualitative.

It should be noted that NH_4_^+^–N apparent removal in this system represents apparent aqueous-phase removal rather than complete nitrogen recovery. Part of the NH_4_^+^ may have been incorporated into MAP, whereas another part could have been converted to NH_3_ and lost through volatilization or gas stripping under alkaline cathodic conditions. Since volatilized NH_3_ was not quantitatively captured, the nitrogen mass balance remains incomplete.

Furthermore, because the current proof-of-concept experiments were conducted using synthetic wastewater, the practical viability of this technology needs to be further evaluated. The suitability of farm, municipal, and industrial waste streams for struvite recovery is feedstock-dependent, and specific supplementation or pretreatment may be required [45]. Real high-strength effluents typically contain complex competing ions, heavy metals, and organic matter that could significantly influence magnesite dissolution, crystallization selectivity, electrode performance, and recovered-product quality. Therefore, future work should focus on NH_3_ capture, real wastewater validation, long-term electrode stability, magnesite utilization efficiency, and the agronomic safety of the recovered products.

## Figures and Tables

**Table 1:** Major components of raw magnesite and the recovered product determined by X-ray fluorescence spectroscopy (XRF) (wt%).

Raw magnesite							
MgO	SiO_2_	CaO	Al_2_O_3_	Fe_2_O_3_	P_2_O_5_	Na_2_O	Cl
72.110	19.232	3.491	3.123	1.329	0.212	0.195	0.081
Recovered product							
P_2_O_5_	MgO	CaO	Na_2_O	SO_3_	K_2_O	ZnO	SiO_2_
47.323	26.234	11.059	8.643	5.501	0.354	0.215	0.199

(Values expressed in oxide form were generated by the XRF software (PCEDX–Pro) on an oxide-equivalent basis and do not indicate the presence of discrete free oxide phases. In particular, P_2_O_5_ represents total phosphorus expressed as P_2_O_5_ equivalent rather than free P_2_O_5_ in the samples. Only the major XRF-reported components are listed).

**Table 2:** Coded levels of the independent variables.

Factor	Level		
	−1	0	1
A—Initial pH	5	7	9
B—Current density (A/m^2^)	22.74	27.29	31.83
C—Initial phosphorus concentration (mM)	6	8	10
D—Initial N:P molar ratio	1	1.5	2

## Data Availability

The original contributions presented in this study are included in the article and Supplementary Material. Further inquiries regarding the data can be directed to the corresponding authors.

## Supplementary Materials

The following supporting information can be downloaded at: https://www.mdpi.com/article/10.3390/molecules31173047/s1, Table S1. Box–Behnken experimental design and response values. The table presents the experimental conditions and measured responses for the response surface methodology (RSM) optimization. Table S2. Summary of ANOVA results for the response surface models. Table S3. Predicted responses and experimental validation under optimized conditions.
