# Correction: Wang et al. Electrochemical Activation of Natural Magnesite for Phosphorus Recovery from Wastewater via In Situ Struvite Crystallization. *Molecules* 2026, *31*, 3047

**DOI:** 10.3390/molecules31203712

**Published:** 2026-10-16

**Authors:** Zepeng Wang, Peidong Su, Chunhui Zhang, Weilong Zhou, Xiaoping Zhong, Guifeng Zhao, Zaining Li, Bingxu Quan

**Affiliations:** School of Chemical & Environmental Engineering, China University of Mining & Technology (Beijing), Beijing 100083, China; 15130208699@163.com (Z.W.); zhou138104@163.com (W.Z.); zxp15779091097@163.com (X.Z.); zgfwor@163.com (G.Z.); zainingli@163.com (Z.L.); 15030531019@163.com (B.Q.)

## Error in Figure

In Figure 1a of the original publication [1], the bar representing the Mg^2+^ concentration at an anodic current density of 0 A/m^2^ was incorrectly plotted. This bar has been corrected to 6.1 mg/L, consistent with the value reported in Section 2.1. The x-axis titles in Figure 1a,b have been updated to “Anodic current density (A/m^2^)”. The y-axis titles in Figure 1a and Figure 1b have been updated to “Concentration (mg/L)” and “Near-cathode pH”, respectively. These corrections do not alter the observed trends of increasing Mg^2+^ concentration in solution and near-cathode pH with increasing anodic current density. The corrected Figure 1 appears below.

During the preparation of Figure 6b, a previously established plotting template was reused [1]. However, the x-axis category labels linked to the template were inadvertently not updated. Consequently, the values “4, 6, 8, and 10” from a previous dataset were retained instead of the correct current-density values of 18.19, 22.74, 27.29, and 31.83 A/m^2^. 

In addition, during the final preparation of the figure, the data links corresponding to the first two groups of mass-fraction values in the template were not fully updated, resulting in some values in the published figure not being retrieved from the final verified dataset. After identifying this issue, we carefully rechecked the original experimental records, calculation sheets, and plotting files, and regenerated Figure 6b directly from the verified source data.

The corrected figure accurately presents the relative mass fractions of cathode-attached deposits and suspended precipitates under the four investigated current densities. This correction does not alter the overall trend reported in the paper, namely that the fraction of cathode-attached deposits decreases markedly with increasing current density, whereas the fraction of suspended precipitates increases. Therefore, the interpretation of the effect of current density and the main conclusions of the paper remain unchanged. 

## Text Corrections

In Section 2.2.3, “Effect of Initial Phosphorus Concentration on NH_4_^+^–N Apparent Removal and PO_4_^3−^–P Recovery”, Paragraph 1, the PO_4_^3−^–P recovery reported for the 8 mM group was the highest replicate value rather than the mean. The two sentences have been corrected to report the mean recovery and the revised phosphate concentration decreases:

However, increasing the initial phosphorus concentration to 8 mM still maintained relatively high recovery performance, with a final NH_4_^+^–N apparent removal of approximately 45–46% and a mean final PO_4_^3−^–P recovery of 53.58%. More importantly, although the percentage recovery at 8 mM was lower than that at 4 mM, the corresponding phosphate concentration decrease was substantially greater, increasing from approximately 2.55 mM at 4 mM to 4.29 mM at 8 mM.

The reagent list in Section 3.1, “Materials and Chemicals”, Paragraph 2, incorrectly included disodium ethylenediaminetetraacetate (EDTA-2Na). This paragraph has been corrected to read:

Ammonium chloride (NH_4_Cl), disodium hydrogen phosphate (Na_2_HPO_4_), sodium sulfate (Na_2_SO_4_), hydrochloric acid (HCl) and sodium hydroxide (NaOH) were of analytical grade and purchased from Shanghai Aladdin Reagent Co., Ltd. (Shanghai, China). Deionized water was used in all experiments.

In Section 3.2, “Experimental Setup”, Paragraph 1, the dimensions of the cylindrical electrolytic cell were incorrectly described in terms of length, width, and height. The first sentence has been corrected to read:

Electrochemical experiments were performed in a custom-made cylindrical plexiglass electrolytic cell with an internal diameter of 10 cm, an internal height of 12 cm, and a working volume of 0.800 L.

In Section 3.4.1, “Water Quality Analysis”, Paragraph 1, the method used to determine Mg and Ca concentrations was incorrectly described as EDTA titration with Mg calculated by difference. The concentrations were measured by ICP-OES. The paragraph has been corrected to read:

NH_4_^+^–N was determined by Nessler’s reagent spectrophotometry according to HJ 535-2009, and PO_4_^3−^–P was measured by the molybdenum blue–ascorbic acid spectrophotometric method according to GB/T 11893-1989. Both measurements were conducted using a UV–Vis spectrophotometer (T6 New Century, Beijing Purkinje General Instrument Co., Ltd., Beijing, China). Mg and Ca concentrations were determined by inductively coupled plasma optical emission spectrometry (ICP-OES; ICPE-9820, Shimadzu, Japan).

In Section 3.4.3, “Evaluation of Nutrient Removal, Cathodic Deposition, and Apparent MAP Purity”, item (2), the heading incorrectly referred to struvite, although the calculation describes the fraction of all recovered solids deposited on the cathode. The heading has been corrected to read:

(2) Fraction of recovered solids deposited on the cathode

In Section 3.4.3, “Evaluation of Nutrient Removal, Cathodic Deposition, and Apparent MAP Purity”, item (3), “Apparent MAP purity”, Paragraph 1, the sample preparation procedure was incorrectly described as scraping deposits from the cathode and dissolving a subsample. The procedure used the cathode-attached deposits and the solids recovered from the bulk solution together. The first two sentences of this paragraph have been replaced by the following three sentences:

The dry mass of cathode-attached deposits was determined from the difference between the dry cathode masses before and after reaction. Solids from the bulk solution were collected by filtration, washed with deionized water, dried, and weighed. To determine the apparent MAP purity of the combined recovered deposits, the cathode bearing the deposits and the dried bulk solids were subjected to acid treatment together using 100 mL of 5% HCl to dissolve the recovered solids.

In the same item, the definition of W_1_ following Equation (12) did not specify the combined recovered solids. The definition has been corrected to read:

W_1_ is the total dry mass of the cathode-attached deposits and the solids recovered from the bulk solution (mg), excluding the mass of the cathode substrate.

The authors state that the scientific conclusions are unaffected. This correction was approved by the Academic Editor. The original publication has also been updated.

## Figures and Tables

**Figure 1:**
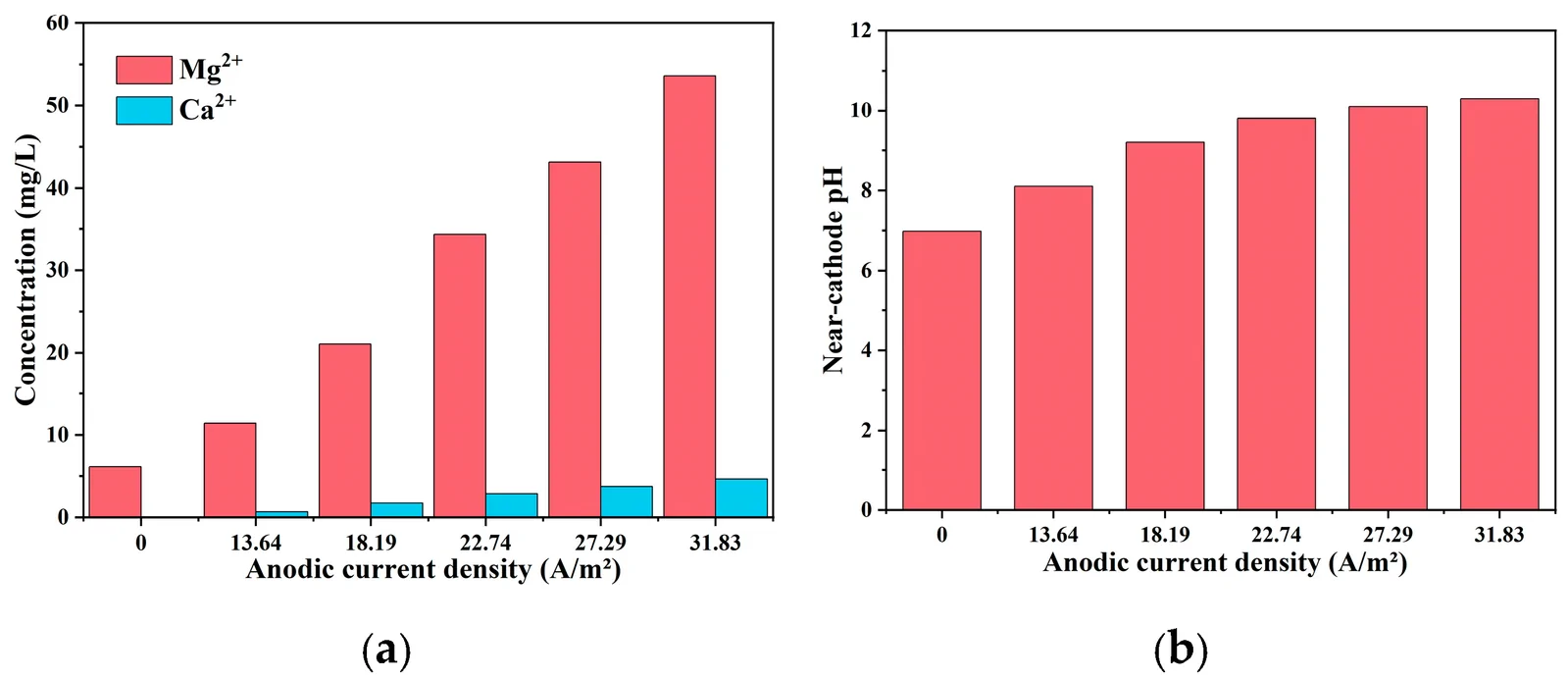
Effects of applied current density on (**a**) Ca^2+^ and Mg^2+^ concentrations in solution and (**b**) pH near the cathode.

**Figure 6:**
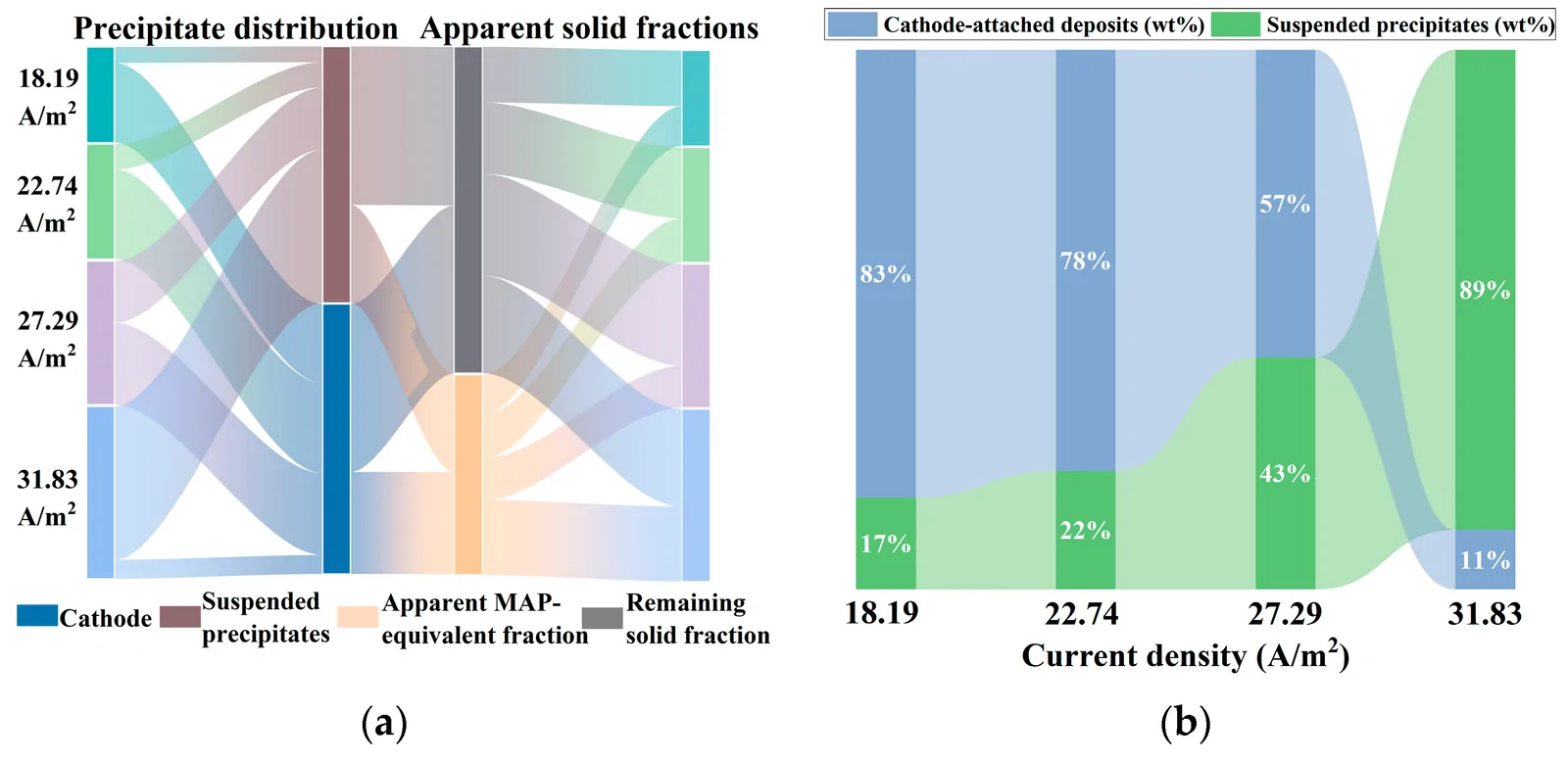
Effects of current density on (**a**) precipitate distribution and apparent MAP-equivalent and remaining solid fractions and (**b**) the relative mass fractions of cathode-attached deposits and suspended precipitates. The remaining solid fraction was calculated by difference.

## References

[1] Wang Z., Su P., Zhang C., Zhou W., Zhong X., Zhao G., Li Z., Quan B. (2026). Electrochemical Activation of Natural Magnesite for Phosphorus Recovery from Wastewater via In Situ Struvite Crystallization. Molecules.

