# Supplementary material for: Electrochemical Activation of Natural Magnesite for Phosphorus Recovery from Wastewater via In Situ Struvite Crystallization
Source: Molecules. 2026 Aug 30;31(17):3047. doi: 10.3390/molecules31173047 (PMC13567028; doi:10.3390/molecules31173047)
Supplement: Supplementary file 1 [file molecules-31-03047-s001.zip › molecules-4490418-supplementary.pdf]

Supplementary Materials

Table S1. Box–Behnken experimental design and response values. The table presents the experimental conditions and measured responses for the response surface methodology (RSM) optimization.

Run	Initial pH	Initial P (mmol/L)	Initial N (mmol/L)	Current density (A/m²)	N:P	PO_4^{3-}-P recovery (%)	NH_4^+-N removal (%)	MAP purity (%)
1	5	6	9	27.29	1.5:1	49.638	35.075	53.356
2	5	10	15	27.29	1.5:1	38.104	24.284	47.884
3	9	6	9	27.29	1.5:1	55.274	56.013	56.38
4	9	10	15	27.29	1.5:1	43.74	41.422	50.908
5	5	8	12	22.74	1.5:1	31.805	25.117	42.218
6	5	8	12	31.83	1.5:1	42.16	41.97	41.302
7	9	8	12	22.74	1.5:1	40.141	41.255	46.342
8	9	8	12	31.83	1.5:1	45.096	63.908	43.226
9	5	8	8	27.29	1:1	35.396	37.982	46.72
10	5	8	16	27.29	2:1	42.794	26.027	47.032
11	9	8	8	27.29	1:1	42.532	57.02	50.344
12	9	8	16	27.29	2:1	46.93	45.065	49.456
13	7	6	9	22.74	1.5:1	51.795	33.335	55.352
14	7	6	9	31.83	1.5:1	57.15	53.088	51.936
15	7	10	15	22.74	1.5:1	37.961	20.643	48.48
16	7	10	15	31.83	1.5:1	47.916	40.396	47.864
17	7	6	6	27.29	1:1	54.486	46.5	59.134
18	7	6	12	27.29	2:1	58.684	36.845	57.946
19	7	10	10	27.29	1:1	41.252	36.109	52.762
20	7	10	20	27.29	2:1	48.85	21.853	53.374
21	7	8	8	22.74	1:1	36.503	35.292	47.966
22	7	8	16	22.74	2:1	42.401	23.336	47.678
23	7	8	8	31.83	1:1	44.159	54.495	45.95
24	7	8	16	31.83	2:1	50.056	42.539	45.662
25	7	8	12	27.29	1.5:1	53.4	45.374	64.08
26	7	8	12	27.29	1.5:1	53.82	45.104	64.48
27	7	8	12	27.29	1.5:1	53.51	45.544	64.39
28	7	8	12	27.29	1.5:1	53.74	45.024	64.18
29	7	8	12	27.29	1.5:1	53.46	45.324	64.45

Table S2. Summary of ANOVA results for the response surface models.

Response	Model F-value	Model p-value	Lack of Fit F-value	Lack of Fit p-value
NH_4^+-N removal	7267.69	<0.0001	0.684	0.7151
PO_4^{3-}-P recovery	2066.31	<0.0001	1.67	0.3277
MAP purity	5683.39	<0.0001	0.4022	0.8893

Table S3. Predicted responses and experimental validation under optimized conditions.

Response	Predicted value	Experimental validation
NH_4^+-N removal (%)	53.01	52.26 ± 0.42
PO_4^{3-}-P recovery (%)	58.68	57.56 ± 0.76
MAP purity (%)	63.36	62.56 ± 0.55
